# TGFβ3, dibutyryl cAMP and a notch inhibitor modulate phenotype late in stem cell-derived dopaminergic neuron maturation

**DOI:** 10.3389/fcell.2023.1111705

**Published:** 2023-02-01

**Authors:** Shanti Sibuea, Joan K. Ho, Colin W. Pouton, John M. Haynes

**Affiliations:** ^1^ Stem Cell Biology Group, Monash Institute of Pharmaceutical Sciences Monash University, Parkville, VIC, Australia; ^2^ National Agency of Drug and Food Control, Jakarta, Indonesia

**Keywords:** Parkinson’s disease, human embryonic stem cells (hESCs), midbrain dopaminergic neurons, dibutyryl cAMP, transforming growth factor–beta, DAPT (PubChem: 5311272)

## Abstract

The generation of midbrain dopaminergic neurons (mDAs) from pluripotent stem cells (hPSC) holds much promise for both disease modelling studies and as a cell therapy for Parkinson’s disease (PD). Generally, dopaminergic neuron differentiation paradigms rely on inhibition of smad signalling for neural induction followed by hedgehog signalling and an elevation of β-catenin to drive dopaminergic differentiation. Post-patterning, differentiating dopaminergic neuron cultures are permitted time for maturation after which the success of these differentiation paradigms is usually defined by expression of tyrosine hydroxylase (TH), the rate limiting enzyme in the synthesis of dopamine. However, during maturation, culture media is often supplemented with additives to promote neuron survival and or promote cell differentiation. These additives include dibutyryl cyclic adenosine monophosphate (dbcAMP), transforming growth factor β3 (TGFβ3) and or the γ-secretase inhibitor (DAPT). While these factors are routinely added to cultures, their impact upon pluripotent stem cell-derived mDA phenotype is largely unclear. In this study, we differentiate pluripotent stem cells toward a dopaminergic phenotype and investigate how the omission of dbcAMP, TGFβ3 or DAPT, late in maturation, affects the regulation of multiple dopaminergic neuron phenotype markers. We now show that the removal of dbcAMP or TGFβ3 significantly and distinctly impacts multiple markers of the mDA phenotype (*FOXA2, EN1, EN2, FOXA2, SOX6*), while commonly increasing both *MSX2* and *NEUROD1* and reducing expression of both *tyrosine hydroxylase* and *WNT5A*. Removing DAPT significantly impacted *MSX2, OTX2, EN1,* and *KCNJ6.* In the absence of any stressful stimuli, we suggest that these culture additives should be viewed as mDA phenotype-modifying, rather than neuroprotective. We also suggest that their addition to cultures is likely to confound the interpretation of both transplantation and disease modelling studies.

## Introduction

Parkinson’s disease (PD) is a progressive nervous system disorder with multiple impacts across the CNS. The characteristic motor impairments of PD, including bradykinesia, rigidity, and resting tremor, have been linked to the loss of the A9 dopaminergic neurons of the substantia nigra pars compacta. Midbrain dopaminergic (mDA) neurons offer promise for both Parkinson’s disease treatment and disease modelling. As a consequence, a number of protocols for differentiating mDAs have been proposed, all with a basic approximation of the embryonic developmental processes (Fasano et al., 2010; Kriks et al., 2011; Takazawa et al., 2012; Qi et al., 2017). Thus, neural induction is followed by midbrain patterning using sonic hedgehog (SHH) and Wnt signalling activators/mimetics. The resultant dopaminergic neurons have been shown to efficiently engraft in animal models of Parkinson’s disease (Fasano et al., 2010; Kriks et al., 2011; Qi et al., 2017) as well as provide a platform for studies of disease mechanisms (Haynes et al., 2021). Commonly, the authentication of mDA cultures is reliant upon the expression of a handful of genes that are essential for development and maturation. Thus, early in differentiation, developing cultures should express floor plate markers such as *Foxa2* and *Otx2*, followed by *En1/2* and *Lmx1a* (Nakatani et al., 2010; Aguila et al., 2014; Arenas et al., 2015). As neurons mature, markers such as *Nr4a2*, *Pitx3* and *Th* (Nunes et al., 2003; Volpicelli et al., 2012) and finally, the dopamine transporters *Slc18a2* and *Slc6a3* (Miller et al., 1999; Ásgrímsdóttir and Arenas, 2020) are expressed. In addition, mDA neuron markers such as *Kcnj6, Aldh1a1* and *Sox6,* and *Calb1* and *Otx2* are used to discriminate between substantia nigra and ventral tegmental area dopaminergic neurons, respectively. During maturation, multiple neurotrophic/survival factors are routinely added to the cultures. These additives include brain-derived neurotrophic factor (BDNF) (Binder and Scharfman, 2004), glial cell-derived neurotrophic factor (GDNF) (Rakowicz et al., 2002), dibutyryl cyclic AMP (dbcAMP) (Mena et al., 1995), N-[N-(3,5-difluorophen-acetyl)-l-alanyl]-S-phenylglycine t-butyl ester (DAPT) (Crawford and Roelink, 2007) and transforming growth factor (commonly TGFβ3) (Luo et al., 2016). BDNF and GDNF are neurotrophic factors known to enhance the survival and differentiation of neural progenitor cells toward the dopaminergic phenotype, protect injured nigrostriatal neurons, and stimulate dopamine turnover and release in rescued neurons (Rakowicz et al., 2002; Binder and Scharfman, 2004). DbcAMP, a cell-permeant analogue of cyclic AMP, has been shown to increase the number of Th-positive cells in fetal midbrain cultures without necessarily increasing neuron survival (Mena et al., 1995). Cultures may also contain DAPT, a Notch signalling inhibitor that blocks γ-secretase to promote differentiation of neurons from human embryonic stem cells (Crawford and Roelink, 2007). Previous work has shown that Notch signalling has no function in the specification of mesencephalic dopaminergic neural precursor cells but plays a vital role in regulating their expansion and differentiation into neurons (Trujillo-Paredes et al., 2016). Lastly, TGFβ3 is a member of the transforming growth factor-β superfamily of multifunctional cytokines that is commonly added to cultures, presumably to stimulate dopaminergic neuron survival (Flanders et al., 1998). The signalling is TGFβ receptor-mediated, involving the Smad and p38 mitogen-activated protein kinase pathways (Airaksinen and Saarma, 2002). Multiple lines of evidence suggest signalling of classic neurotrophic factors such as BDNF and GDNF may be affected by TGFβ (Schober et al., 2007; Luo et al., 2016) which also plays a critical role in astrocyte and microglial function under physiological and injury conditions (Bialas and Stevens, 2013; Butovsky et al., 2014). While these factors are routinely added for developing mDA cultures, their ability to affect cultures beyond commonly under-justified, short-term effects on survival is unclear.

In this study, we maintain mature mDA neurons in the presence of GDNF, BDNF, dbcAMP, DAPT, and TGFβ3 and assess the impact that the removal of dbcAMP, DAPT or TGFβ3 has upon mDA neuron culture development. We now show that a loss of dbcAMP downregulates transcripts associated with a neuronal midbrain phenotype (*EN1*, *TH*, *PITX3*, but not *NR4A2; SLC6A3 or SLC18A2*)*.* In contrast, DAPT removal downregulates *MSX2, OTX2, KCNJ6* and *nestin* while upregulating the astrocyte markers *GFAP and S100B.* TGFβ3 removal regulates the expression of early markers (*OTX2, MSX2* and *NEUROD1*), as well as *TH* and the astrocyte marker S100B. In conclusion, we found that culture additives play a role in maintaining what might be construed as a mature mDA phenotype. We speculate that additive-induced promotion of a dopaminergic phenotype may i) mask inefficient patterning and therefore contribute to the equivocal outcomes of transplantation therapies and ii) impact the use of these neurons as disease models.

## Materials and methods

### Human embryonic stem cell culture (hESC)

A modified H9 pluripotent stem cell line where eGFP was expressed under the control of LMX1A promoters (LMX1A-eGFP) (Niclis et al., 2017) was used to track the expression of LMX1A. All plates or flasks were pre-coated with 0.5 µg/cm^2^ Laminin-521 (Life Technologies, Australia). Undifferentiated cells were grown using a slightly modified method of (Watmuff et al., 2015). Briefly, pluripotent stem cells were seeded at a density of 3,000 cells per cm^2^ and cultivated in Essential 8 Medium with Essential 8 supplement (Thermo Fisher Scientific, Australia) and Penicillin/Streptomycin (Life Technologies, Australia) at 37°C in a humidified incubator containing 5% CO_2_. Cells were passaged at ∼80% confluence (ReLeSR™, Stemcell technologies, Australia). To enhance the survival of cells seeded at low-density, the ROCK inhibitor (10 μM Y-27632) was added to the medium for the first 24 h after passaging. Cells were maintained for a maximum 10 passages.

### Differentiation/neural induction

On day one of differentiation, the culture medium was replaced with differentiation medium 1 (D1): Knockout DMEM (Life Technologies, Australia), 15% knockout serum replacement (KSR, Life Technologies, Australia), 1% MEM Non-Essential Amino Acids Solution (Life Technologies, Australia), 2 µM GlutaMAX-I (Life Technologies, Australia), 100U/mL Penicillin/Streptomycin (Life Technologies, Australia) and 0.1 mM β-mercaptoethanol (Life Technologies, Australia) on Matrigel™ (BD Biosciences, Australia) until fully confluent (around 1.5 × 10^5^ cells/cm^2^). From day five of differentiation D1 was gradually changed to differentiation medium 2 (D2): DMEM: F12 (Life Technologies, Australia), N_2_ supplement (Life Technologies, Australia), 100U/mL Penicillin/Streptomycin, 50 mg/mL human apo-transferrin (Sigma Aldrich) and 0.48 mg/mL human insulin (Sigma Aldrich). The change in media occurred over 6 days, with the D1:D2 ratios of 3:1 (days five and six), 1:1 (days seven and eight) and 1:3 (days nine and ten). From day 11 onwards, media was changed to neurobasal medium (NBM): Neurobasal^®^ medium (Thermo Fisher Scientific, Australia); B27 supplement (Life Technologies, Australia); 100 U/mL Penicillin/Streptomycin; 2 mM GlutaMAX-I; 20 ng/mL recombinant GDNF (Lonza Peprotech, Australia), 20 ng/mL recombinant BDNF (Lonza Peprotech, Australia), 200 nM ascorbic acid (Sigma Aldrich, Australia). Specific cultures also contained combinations of 10 nM DAPT (Jomar Life Research, Australia), 0.5 mM dbcAMP (Sigma Aldrich, Australia) and 1 ng/mL recombinant TGF-β3 (Lonza Peprotech, Australia). Additive removal occurred from Day 40 onwards.

### Immunocytochemistry

On day 65 of differentiation, cells were fixed with 4% paraformaldehyde and permeabilized with permeabilization solution 1 containing 0.1% (v/v) x-100, 0.1% (v/v) Tween-20, 20% (v/v) Hybri-Max dimethylsulphoxide (DMSO) in DPBS without calcium or magnesium (DPBS −/−). Then further incubated with permeabilization solution 2 containing 0.1% (v/v) Tween-20, 0.1% (v/v) Triton X-100, 0.1% deoxycholate, 0.1% tergitol solution, 20% (v/v) DMSO in DPBS (−/−). Cells were blocked with 3% (v/v) donkey serum in permeabilization solution 2 following incubation with primary antibody rabbit anti-PITX3 (1:100 Invitrogen), sheep anti-TH (1:500 Abcam), goat anti-GIRK2 (1:100 Abcam), rabbit anti-WNT5A (1:200 Abcam), mouse anti-TUBB3/chicken anti-MAP2 (1:1000 Abcam) in 3% donkey serum (1:100 dilution) overnight at 4°C. Then cells were stained with a secondary antibody (Supplementary Table S1) in 3% donkey serum (1:1000 dilution) for 4 h at room temperature. On the day of imaging, cells were stained with DAPI (1:5000 dilution) in DPBS (−/−). Fluorescence was detected using a Nikon A1R Confocal Microscope.

### Reverse transcriptase quantitative polymerase chain reaction (RT-qPCR)

On day 65 of differentiation, total RNA was extracted from cultures using the Bioline RNA Micro Kit (Bioline, Australia) according to the manufacturer’s instructions. Samples were analyzed for RNA content using a Nanodrop ND-1000 (Thermo Fisher, Australia) spectrophotometer. RNA samples were converted to cDNA using SensiFAST™ cDNA Synthesis Kit (Bioline, Australia). cDNA samples were then diluted with an appropriate amount of DNase RNAse-free water and TaqMan^®^ probes and mixed with SensiFAST Probe No-ROX Kit. RT-qPCR was performed in triplicate using the CFX96 Real-Time PCR detection system. HPRT1 and TBP were used as reference genes. Relative gene expression was expressed as target gene Ct values to reference genes. A list of Taqman probes is shown in Supplementary Table S2, while Supplementary Table S3 shows delta delta Ct values for each replicate.

### Western blotting

Cells were lysed in RIPA lysis buffer (Thermo Fisher, Australia). Protein concentrations were determined by BCA assay (Thermo Fisher, Australia). Then, 20 ug protein was resolved using 4%–12% SDS polyacrylamide gel and transferred to a PVDF membrane (Thermo Fisher, Australia). The membranes were incubated overnight at 4 °C in a blocking solution containing 5% (w/v) non-fat dry milk in PBS with 0.1% Tween-20. Subsequently, membranes were incubated with primary antibodies, followed by an incubation either with fluorescence or horseradish peroxidases-secondary antibody using SuperSignal West Pico Chemiluminescent Substrate (Pierce, Thermo Fisher). GAPDH was used as an internal loading control. Detection of fluorescence was performed by Amersham™ Typhoon™ Biomolecular Imager (GE Healthcare) and HPR was performed by Gel Doc TM (BioRad). Quantitative data analysis was performed using ImageJ. All bands were normalized to GAPDH expression.

### Cell thresholding

Cell fluorescence was thresholded using ImageJ (Supplementary Table S4) to enable estimations of cell protein fluorescence: DAPI fluorescence ratios.

### Transcription factor binding sites

Using MotifMap (Daily et al., 2011), we identified potential gene regulatory motifs, an arbitrary 10,000 base pairs up and 2000 base pairs downstream of the transcription start sites with FDR ≤0.5. We sorted the list of putative binding sites by the number of sites (the highest number came first in the list) and analyzed the common transcription putative binding site of several genes associated with mDA development whose expression is affected by additives added during mDA neuron maturation (Supplementary Table S5).

### Statistical analysis

Unless otherwise stated, results from experiments are presented as the mean ± standard error of the mean (SEM) of at least three biological replicate experiments (each with at least two technical replicates). Statistical analysis was performed on RT-qPCR raw data with a one-sample *t*-test, or One-way ANOVA, followed by *post hoc* Dunnett’s test to show effects *versus* control for immunocytochemistry results. All analyses were performed using PRISM v8.00 (GraphPad Software, CA, United States). In all cases, a *p* < 0.05 was considered significant.

## Results

### Additives and mDA phenotype

We studied the effect of the common tissue culture additives dbcAMP, TGFβ3 and DAPT upon the regulation of key dopaminergic neuron phenotype markers of early and late development, as well as A9 subtype mDA neuron specification. We maintained mature mDA neurons in “normal” media (i.e., in the presence of GDNF, BDNF, dbcAMP, DAPT, and TGFβ3) and assessed, at day 65 of differentiation, the impact that removal of dbcAMP, DAPT or TGFβ3 had upon genes expressed early in dopaminergic development (*FOXA2, MSX2, LMX1A*), in maturing (*PITX3, NR4A2, TH*) and mature cultures (*SLC18A2, SLC6A3*). We also looked for changes to the general character of the cultures (*nestin, TUBB3*), astrocyte markers (*GFAP, S100B*) and endogenous WNTs (*WNT1, WNT5A*).

### dbcAMP and mDA phenotype

**FIGURE 1:**
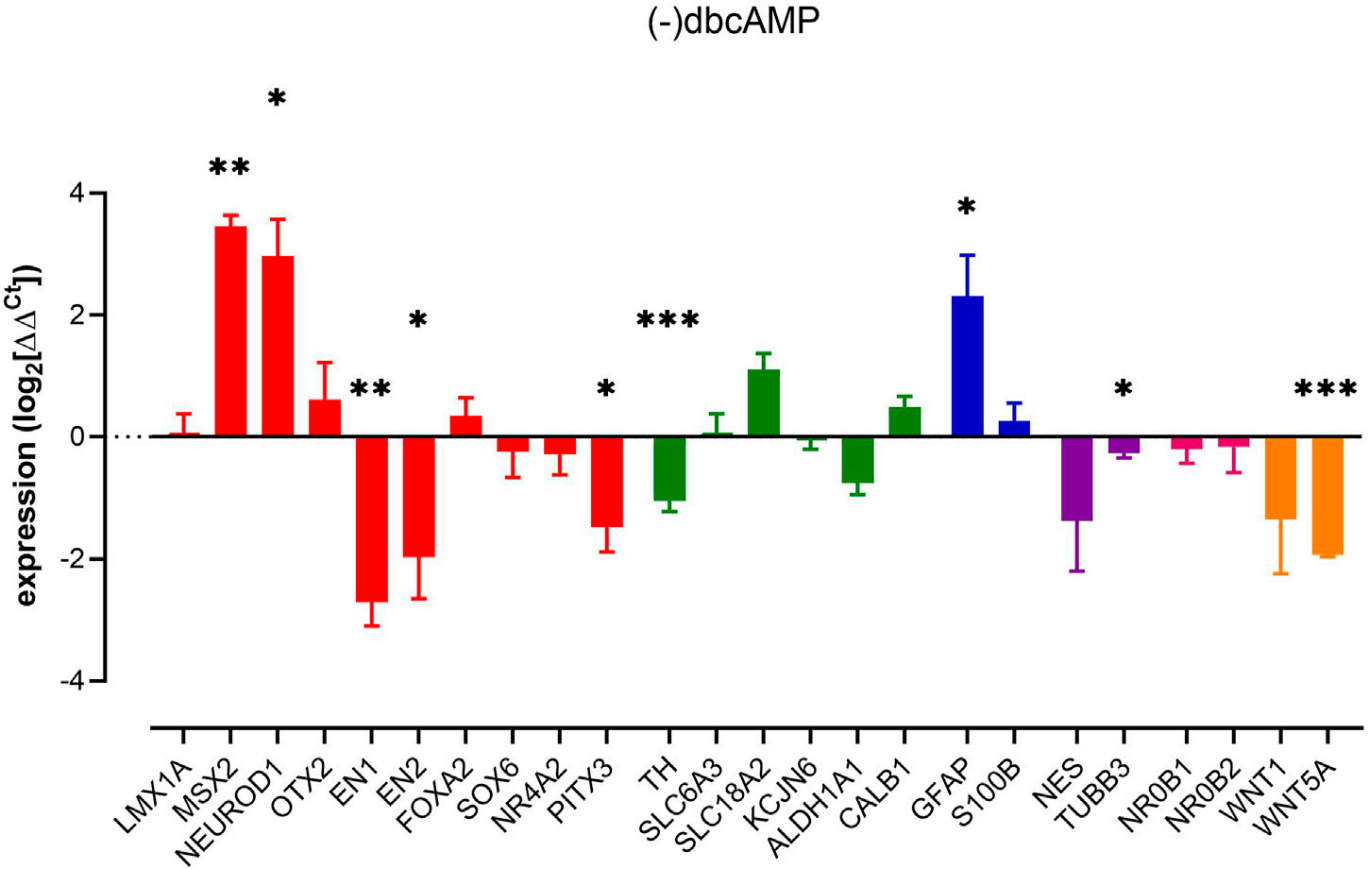
Impact of removal of dbcAMP upon pluripotent stem cell-derived dopaminergic neuron cultures. Columns show mean (+s.e. mean) data normalized to HPRT1 and TBP and expressed as fraction of (+) dbcAMP control (from matched differentiations). Statistical analysis was *via* one sample t-tests (*n* = 3–6 biological replicates). Graphs show mean +/- s. e. mean changes; * = *p* < 0.05, ** = *p* < 0.01 and *** = *p* < 0.001.

The absence of dbcAMP, a membrane-permeable analogue of cyclic-AMP, promoted significant changes across dopaminergic neuron-containing cultures. Notably, decreased expression of a number of markers associated with dopaminergic neuron development and maturation, including *EN1, EN2, PITX3*, and most notably, *TH,* with no change in *NR4A2, SLC6A3,* and *SLC18A2* expression. There was also a small decrease in a generic neural marker *TUBB3* (Figure 1).

Given the roles that Wnt1 and Wnt5a play in regulating the genes involved in mDA development (Castelo-Branco et al., 2003; Prakash et al., 2006; Bryja et al., 2007; Joksimovic et al., 2009; Andersson et al., 2013), we assessed the expression of these WNT family ligands, *WNT1* and *WNT5A*. RT-qPCR analysis showed significant downregulation of *WNT5A* (β-catenin-independent) but not β-catenin-dependent WNT1 (Figure 1). That WNT5A can be regulated by removal of dbcAMP likely indicates an endogenous WNT signalling present in mature cultures.

Other interesting observations were the elevations of *MSX2, NEUROD1* and *GFAP* brought on by the removal of dbcAMP. The increase of GFAP, a type III intermediate filament protein present in mature astrocytes (Brenner and Messing, 2021) indicated an increase in reactive gliosis. This is consistent with previous studies showing that many astroglia genes are differentially regulated by cAMP signalling (Horvat and Vardjan, 2019), including the downregulation of reactive astrocyte markers (Paco et al., 2016).

### TGFβ3 and mDA phenotype

**FIGURE 2:**
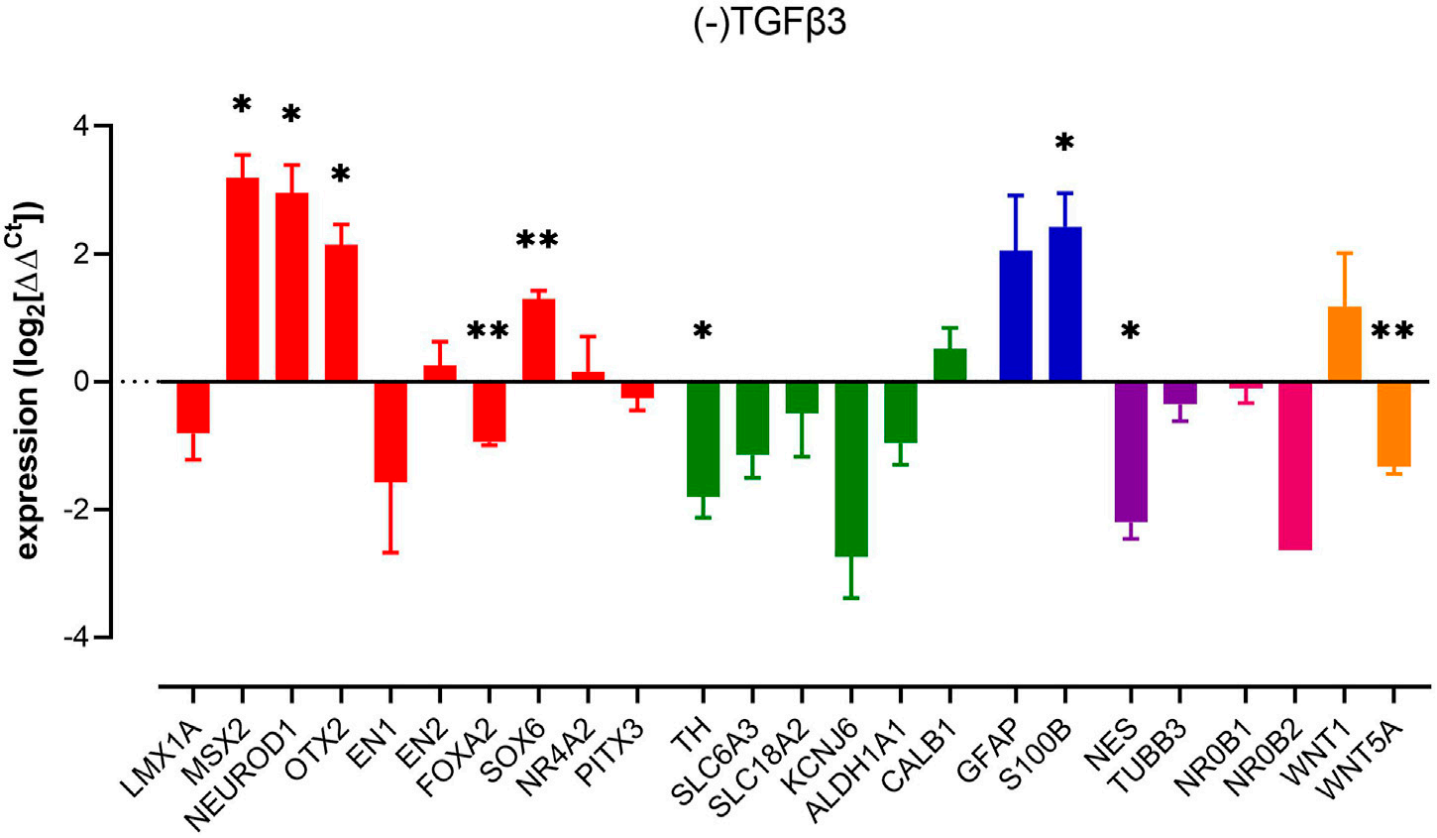
Impact of removal of TGFβ3 upon pluripotent stem cell-derived dopaminergic neuron cultures. Columns show mean (+s.e. mean) data normalized to HPRT1 and TBP and expressed as fraction of (+) TGFβ3 control (from matched differentiations). Statistical analysis was *via* one sample t-tests (*n* = 3 biological replicates). Graphs show mean +/- s. e. mean changes; * = *p* < 0.05, ** = *p* < 0.01 and *** = *p* < 0.001.

Next, we investigated the impact of removal of TGFβ3 from our cultures. We observed changes in several key markers, often, but not exclusively associated, with neuronal-lineage commitment. Thus, we observed upregulations of *nestin, MSX2, NEUROD1, OTX2,* and *SOX6*. In contrast, we observed reduced expression of markers commonly associated with a more committed dopaminergic phenotype, *FOXA2* and *TH* (Figure 2). Interestingly, the astrocyte-related gene *S100B* was increased significantly without an increase in the reactive astroglial marker *GFAP*, possibly indicating astroglial genesis rather than activation. The removal of TGFβ3, similar to the removal of dbcAMP, led to a decrease in both *WNT5A* and *TH* expression and a profound loss of the neural progenitor marker, *nestin* (Figure 2).

### DAPT and mDA phenotype

**FIGURE 3:**
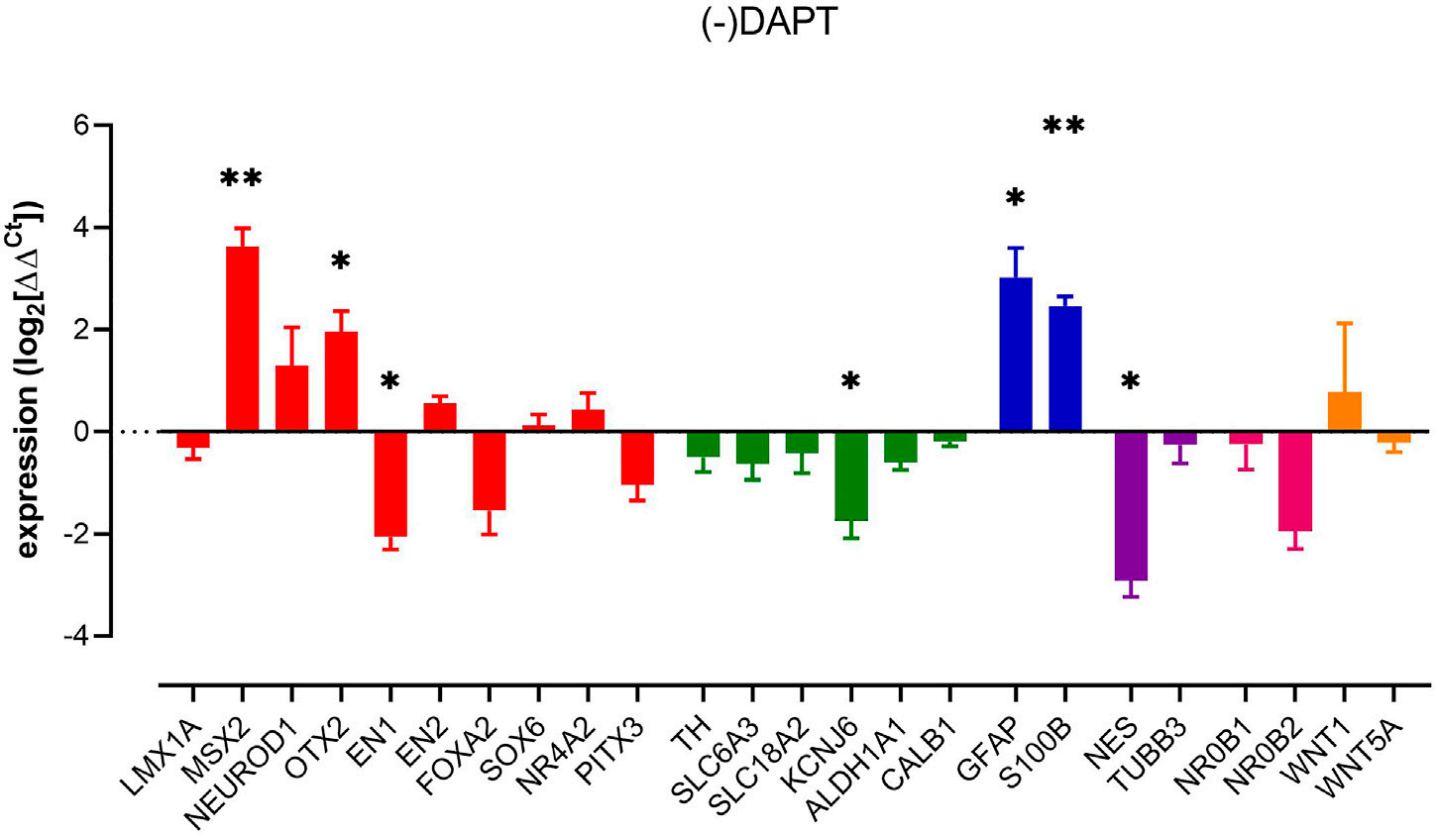
Impact of removal of DAPT upon pluripotent stem cell-derived dopaminergic neuron cultures. Columns show mean (+s.e. mean) data normalized to HPRT1 and TBP and expressed as fraction of (+) DAPT control (from matched differentiations). Statistical analysis was *via* one sample t-tests (*n* = 3 biological replicates). Graphs show mean +/- s. e. mean changes; * = *p* < 0.05, ** = *p* < 0.01, and *** = *p* < 0.001.

The removal of DAPT (activating Notch signalling) increased the expression of *MSX2, OTX2* and astrocyte markers *GFAP* and *S100B*. In contrast, *EN1*, *nestin* and *KCNJ6* showed reduced expression (Figure 3). These data indicate that, just as with the removal of dbcAMP and TGFβ3, the factors that may be construed to be part of the regulatory framework of dopaminergic neuron phenotype can be impacted selectively, and late in differentiation.

Increased expression of *GFAP* and *S100B* in our culture was anticipated as the Notch pathway has a prominent role in controlling neuronal morphology and determining astrocyte fate (Wang et al., 2019). In addition, DAPT suppresses the activation of astrocytes (Wang et al., 2019), (Qian et al., 2019) leading us to believe that notch signalling contributes to astrocyte function in this culture paradigm.

### Protein expression following additive removal

We undertook fluorescence imaging (eGFP) along with immunolabelling and western blotting to establish changes in protein expression.

**FIGURE 4:**
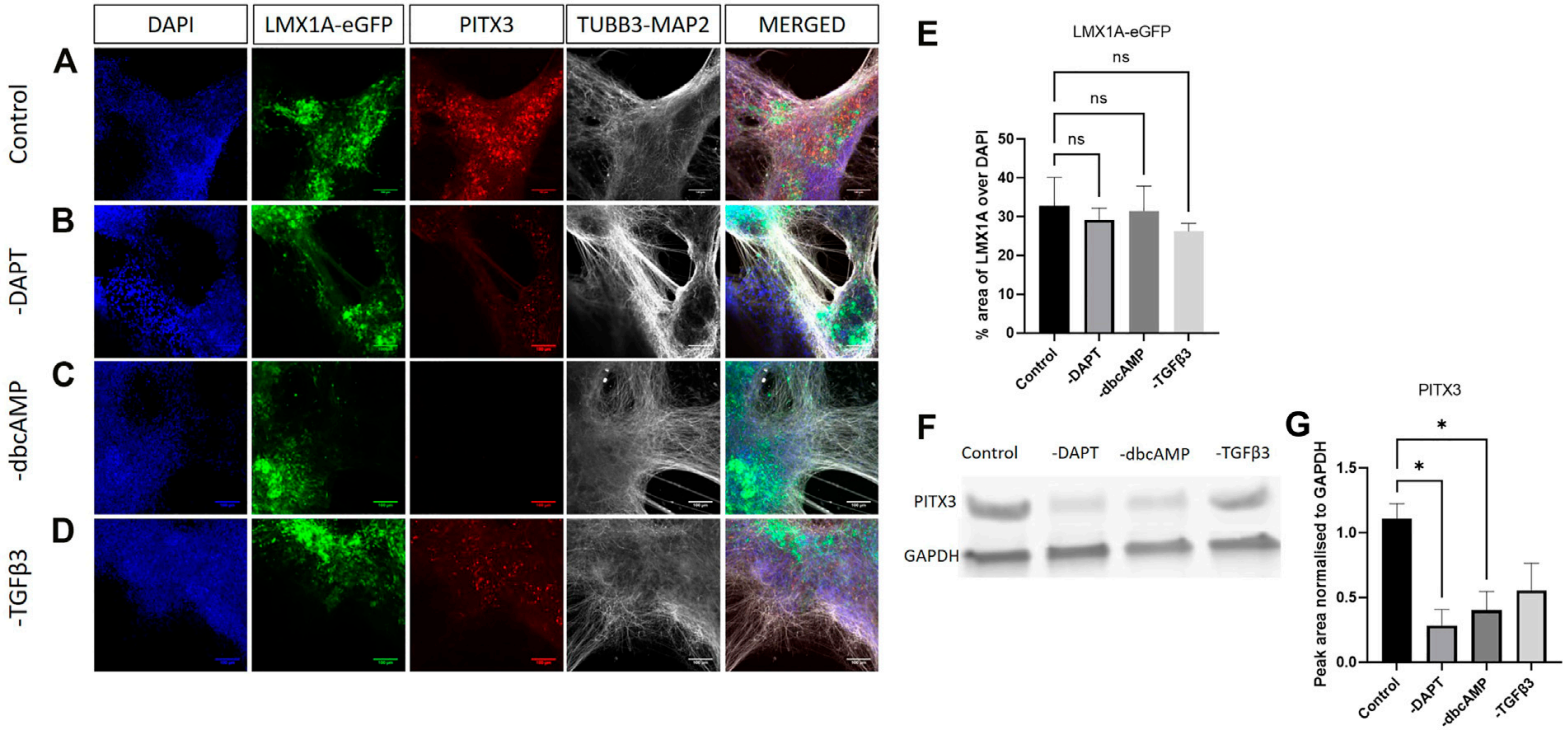
Immunolabelling of PITX3, TUBB3/MAP2 in LMX1A-eGFP cultures. Panel **(A)** shows, from left to right, DAPI nuclear labelling (blue), LMX1A-eGFP fluorescence (green), PITX3 (red) and TUBB3/MAP2 in the same channel (white) and a color combined image (merged). Panels **(B–D)** show the corresponding data, but following cultivation in the absence of DAPT, dbcAMP and TGFβ3, respectively. Scale bar indicates 100 μm. Panel **(E)** shows LMX1A-eGFP fluorescence as a fraction of DAPI fluorescence (*n* = 3). Panel **(F)** shows a typical Western blot while panel G shows changes in PITX3 expression following removal of additives (*n* = 3). Bar graphs show mean +/- s. e. mean; * = *p* < 0.05.

Analysis of LMX1A-eGFP expression showed bright widespread eGFP across cultures, not necessarily localized to TUBB3-MAP2 immunolabelling, and not greatly impacted by removal of DAPT, dbcAMP or TGFβ3 (Figures 4A–D). Quantification of eGFP fluorescence (expressed as a percentage of DAPI fluorescence) showed that, consistent with the RT-qPCR studies, the removal of additives has little impact upon LMX1A-eGFP fluorescence (Figure 4E). In contrast PITX3 protein expression (Figure 4F shows typical blotting) was particularly impacted by removal of DAPT and dbcAMP (Figure 4G).

**FIGURE 5:**
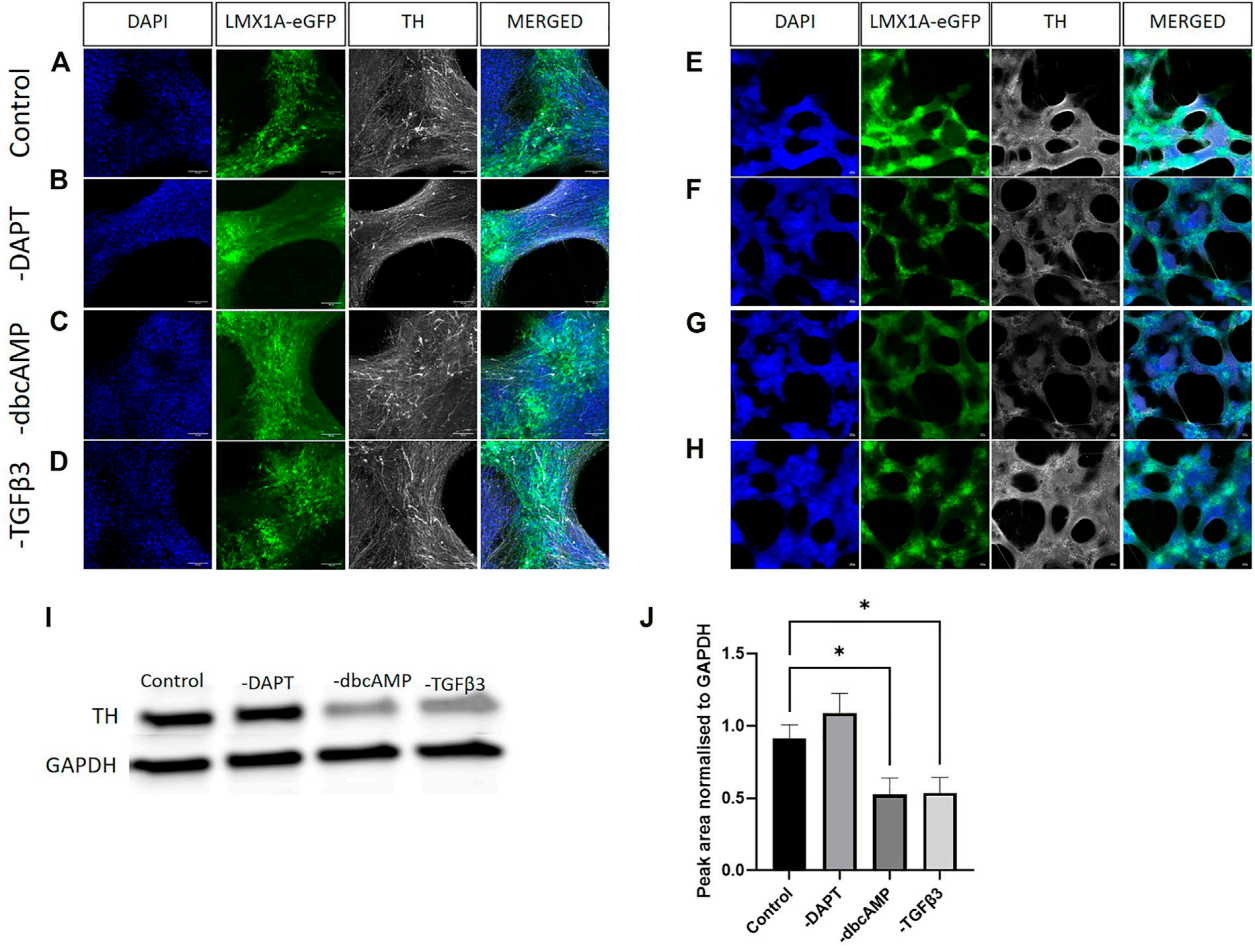
Immunolabelling of tyrosine hydroxylase in LMX1A-eGFP cultures. Panels [**(A–D)**, ×20 objective) show, from left to right, DAPI nuclear labelling (blue), LMX1A-eGFP fluorescence (green), TH (white) in control (panel A), and after removal of DAPT [panel **(B)**], dbcAMP [panel **(C)**] and TGFβ3 [panel **(D)**], respectively. Panels **(E–H)** show corresponding low power images (×4 objective). Scale bar indicates 100 μm. Panel **(I)** shows typical Western blotting, while panel **(J)** shows quantification of TH (*n* = 3) following the removal of additives. Bar graphs show mean +/- s. e. mean; * = *p* < 0.05.

We labelled for TH to observe changes in expression and evidence of colocalization with LMX1A-eGFP positive cells following the removal of culture additives (Figures 5A–H, show lower magnification images of the same fields of view). While the higher magnification images showed, regardless of additive removal, the presence of TH immunofluorescence, low power magnification indicated a widespread reduction of TH across cultures in response to the removal of dbcAMP (Figure 5G). This finding was confirmed using immunoblotting (Figure 5I shows typical labelling) that showed a loss of TH protein across cultures following the removal of dbcAMP and TGFβ3 (Figure 5J).

**FIGURE 6:**
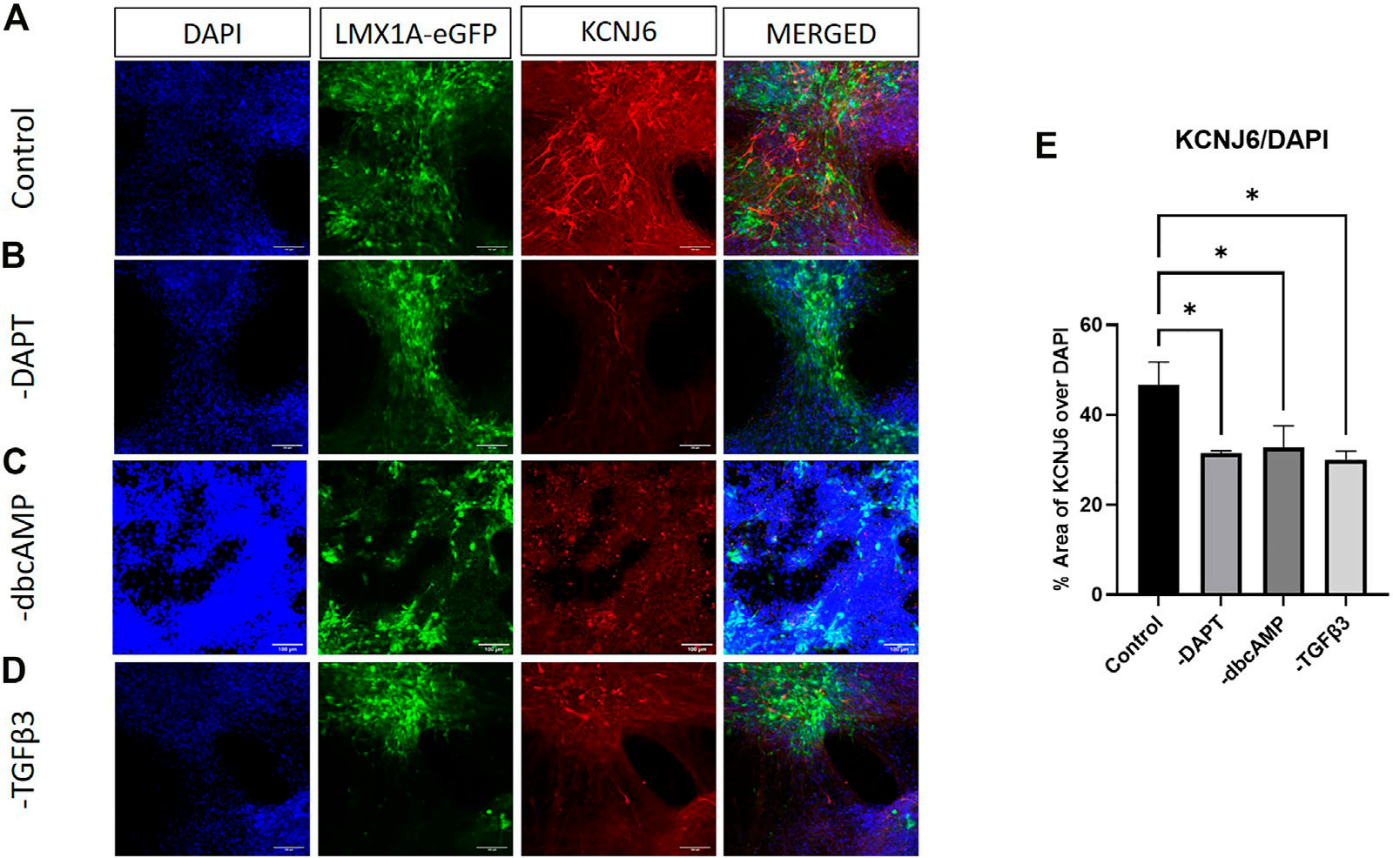
Immunolabelling of KCNJ6 in LMX1A-eGFP cultures. Panel **(A)** shows, from left to right, DAPI nuclear labelling (blue), LMX1A-eGFP fluorescence (green), KCNJ6 (red) and a color combined image (merged). Panels **(B–D)** show the corresponding data, but following cultivation in the absence of DAPT, dbcAMP and TGFβ3, respectively. Scale bar indicates 100 μm. Panel **(E)** shows KCNJ6 fluorescence as a fraction of DAPI fluorescence (*n* = 3). Bar graphs show mean +/- s. e. mean; * = *p* < 0.05.

The widely used marker of A9 phenotype, KCNJ6, showed abundant expression in neuron-like cells across cultures, although the strongest expression, was not particularly colocalized with LMX1A-eGFP (Figure 6A). KCNJ6 expression was, in contrast with RT-qPCR data, impacted by the removal of all additives (Figures 6A–E, shows thresholding analysis of KCNJ6 fluorescence as a fraction of DAPI labelling).

**FIGURE 7:**
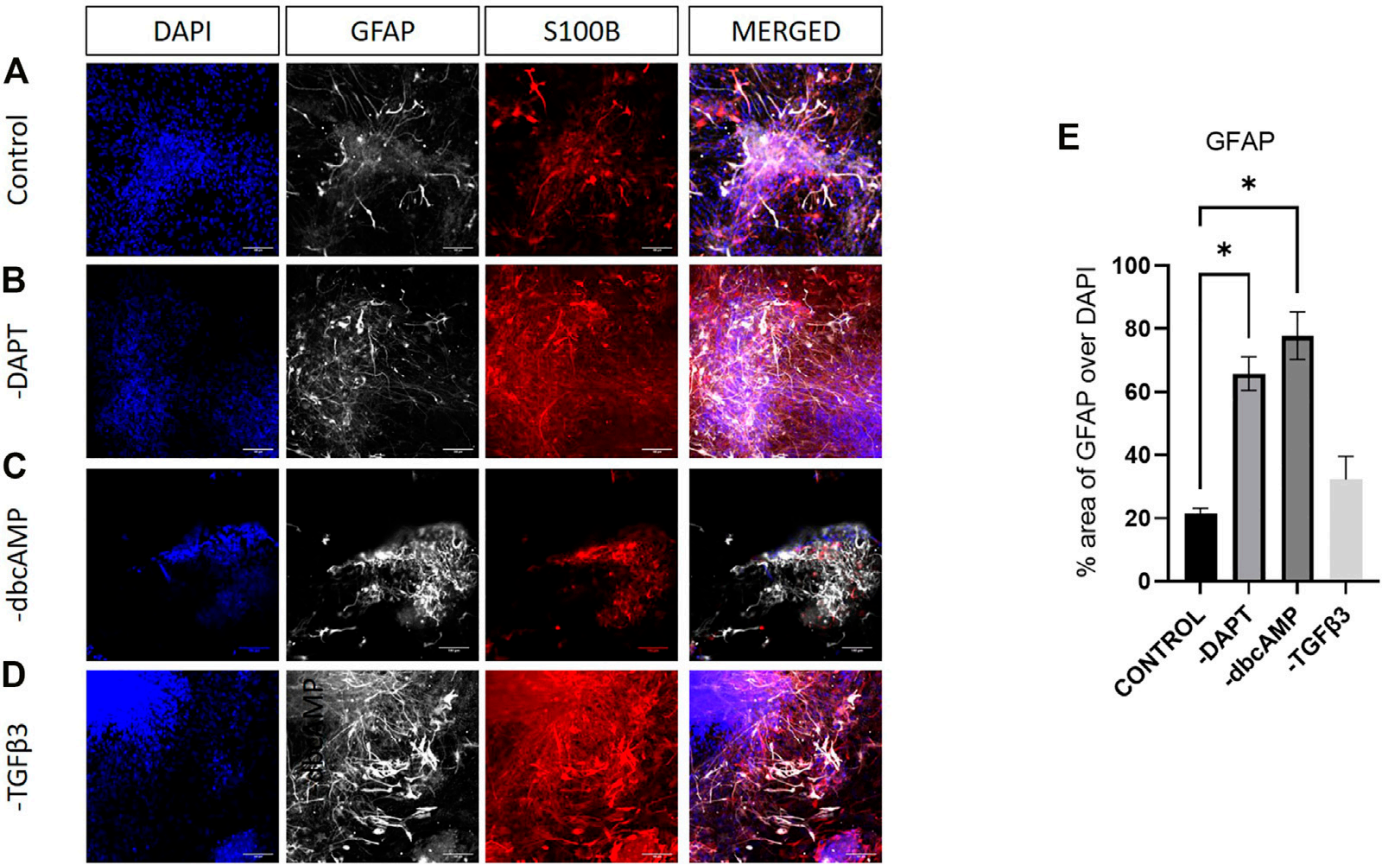
Immunolabelling of GFAP and S100B in LMX1A-eGFP cultures. Panel **(A)** shows, from left to right, DAPI nuclear labelling (blue), GFAP (white), S100B (red) and a color combined image (merged). Panels **(B–D)** show the corresponding data, but following cultivation in the absence of DAPT, dbcAMP and TGFβ3, respectively. Scale bar indicates 100 μm. Panel **(E)** shows GFAP fluorescence as a fraction of DAPI fluorescence (*n* = 3). Bar graphs show mean +/- s. e. mean; * = *p* < 0.05.

GFAP and S100B showed considerable colocalization in cultures, although the most intense GFAP labelling was usually associated with lower levels of S100B expression (Figure 7A). Increases in GFAP in response to the removal of DAPT, dbcAMP or TGFβ3 were clearly evident in cultures (Figures 7B–D). There were widespread elevations of S100B apparent after the removal of DAPT and TGFβ3 (Figure 7E).

**FIGURE 8:**
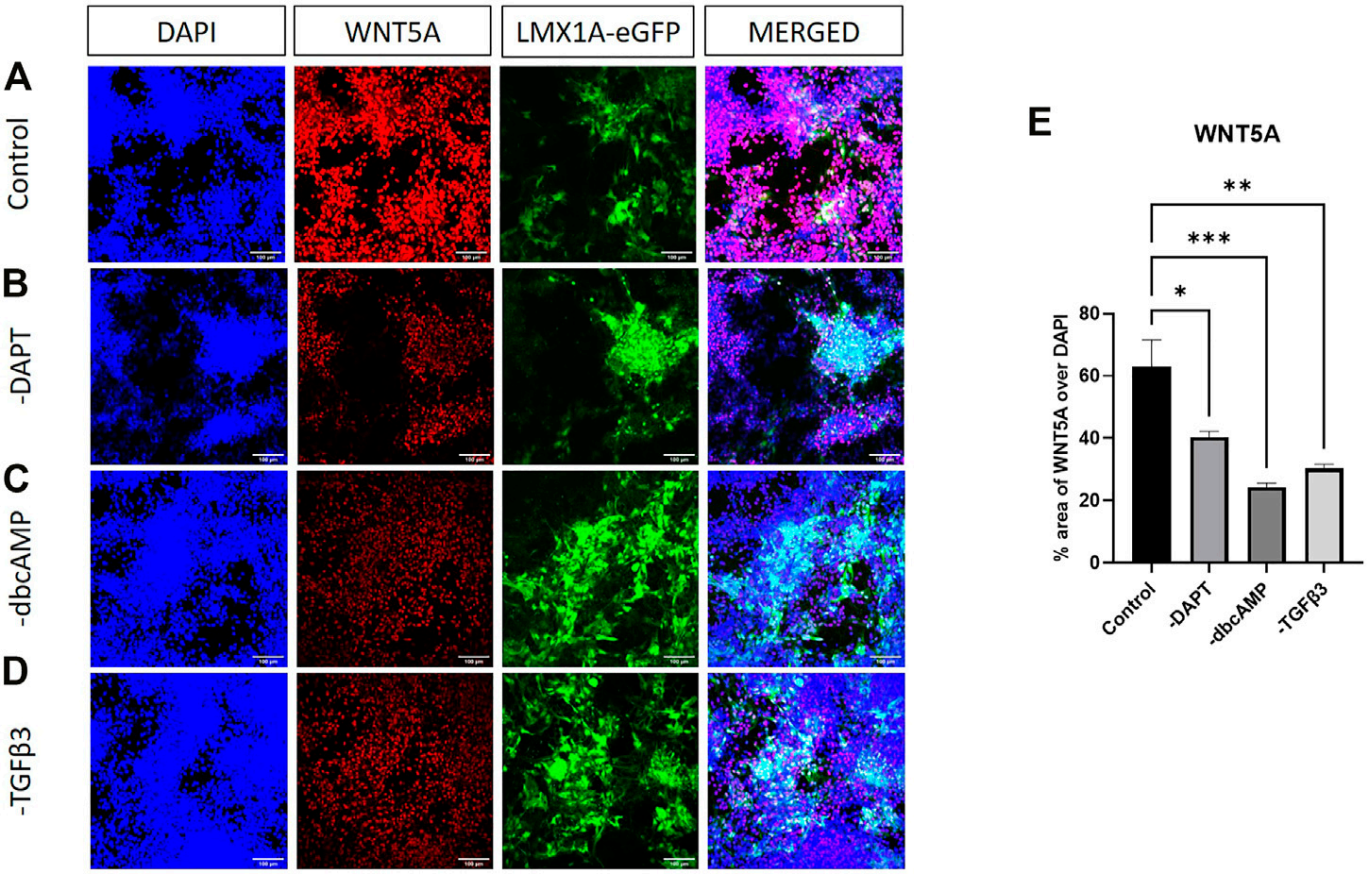
Immunolabelling of WNT5A in LMX1A-eGFP cultures. Panel **(A)** shows, from left to right, DAPI nuclear labelling (blue), WNT5A (red), LMX1A-eGFP fluorescence (green) and a color combined image (merged). Panels **(B–D)** show the corresponding data, but following cultivation in the absence of DAPT, dbcAMP and TGFβ3, respectively. Panel **(E)** shows WNT5A fluorescence as a fraction of DAPI fluorescence (*n* = 3). Scale bar indicates 100 μm. Bar graphs show mean +/- s. e. mean; * = *p* < 0.05, ** = *p* < 0.01, and *** = *p* < 0.001.

Given the modulation of *WNT5A* by removing dbcAMP and TGFβ3, we looked for WNT5A protein in cultures. Surprisingly WNT5A expression was both intense and widespread across cultures, evident in LMX1A-eGFP positive and other cell types, but largely restricted to nuclear regions (Figure 8A). WNT5A expression was clearly reduced following the removal of dbcAMP and TGFβ3 (Figures 8B–D). Quantifying culture-wide fluorescence showed reductions in WNT5A protein expression following the removal of all additives (Figure 8E).

### Transcription factor motif analysis

We turned to MotifMap analysis to investigate candidate regulatory motif sites involved in the changes of transcript observed in the culture. The top five motifs with FDR ≤0.5 (10,000 upstream and 2000 downstream, Supplementary Table S4) ranked by a number of sites revealed common putative transcription factor binding sites associated with the mDA development, namely NEUROD1, MZF 1_4, HNF4A, LEF-1 and YY1. We then searched for motifs mainly involved in the cAMP pathway, the SMAD pathway and the Notch signalling pathway in the hope of revealing candidate motifs affected by the additives. Intriguingly, *SLC18A2* and *NR4A2*, which possess many putative CREB binding sites, seem unaffected by the absence of dbcAMP. Conversely, *EN1* and *PITX3,* which possess few putative CREB binding sites, were most affected. Moreover, none of the genes was affected by the removal of TGFβ3 (e.g. *FOXA2, MSX2, NESTIN, NEUROD1, TH, WNT5A*) which have many putative SMAD binding sites, the main signal transducer for receptors of the transforming growth factor beta superfamily. Similarly, the genes affected by blocking notch signalling, *MSX2, OTX2, KCNJ6, GFAP, S100B,* and *nestin,* possess few putative RBPJ binding sites.

## Discussion

### General characteristics of the cultures

#### Early markers MSX2, NEUROD1, and nestin

The acquisition of functional maturity by postmitotic neurons relies on the complex interactions of multiple intracellular signalling pathways (Lepski et al., 2011). *In vitro*, supplements and growth factors (i.e., dbcAMP, TGFβ3 and DAPT) are driving these pathways to ensure the survival of the neurons and/or accelerate maturation/survival (Kriks et al., 2011; Kirkeby et al., 2012; Arenas et al., 2015). Indeed, our earlier work has shown how these cultures develop over time and the profound impact that dysregulation of WNT signalling has upon phenotype (Haynes et al., 2021). However, in spite of an extensive amount of empirical data, the factors involved in maturation, as well as the general mechanisms regulating the timing of neuronal maturation, are unclear (Takazawa et al., 2012; Telias et al., 2014). In this study, we patterned and cultured mDA conventionally for 40 days prior to the removal particular additives. We then analyzed the expression of early and late markers of neuron and glial identity by RT-qPCR, Western blotting and immunostaining at day 65 of differentiation, when cultures are largely mature (Watmuff et al., 2015).

The most consistent observation following the removal of dbcAMP, TGFβ3 or DAPT was an increase in expression of the transcriptional repressor, *MSX2*. MSX2/*Msx2* (muscle segment homeobox 2) is a homeobox transcription factor of the msh family critical for mesendoderm differentiation. It is a direct target gene of the BMP pathway and can be synergistically activated by Wnt signals *via LEF1* during mesendoderm induction (Wu et al., 2015). *Msx2* is expressed in mouse neural progenitors in the developing ventricular zone (La Manno et al., 2016) in the dopaminergic subset of Lmx1a-positive neural progenitors (Kee et al., 2017). *MSX2* regulates various cellular processes including cellular proliferation and differentiation during embryonic development (Han et al., 2007; Bernal and Arranz, 2018). These observations are consistent with work showing that Notch1 activity inhibits BMP signalling at the level of *Msx2* expression in embryonic cerebellar development (Machold et al., 2007) and that Notch signalling induces direct transcriptional activation of the *MSX2* gene (Shimizu et al., 2009). In the developing nervous system, the Notch pathway has a prominent role in controlling neuronal morphology and determining astrocyte fate (Wang et al., 2019), which directs an irreversible switch from neurogenic to gliogenic activity (Morrison et al., 2000). Such activity may explain the elevations of S100B and GFAP following the removal of DAPT from cultures.

The removal of both dbcAMP and TGFβ3 elevated the expression of *NEUROD1*. NEUROD1 is a key regulator of neuron formation and function, it is expressed from early in mDA development into adulthood (Mesman et al., 2018). Its expression is enhanced directly by forskolin (Paldino et al., 2014) but, in our hands, the loss of dbcAMP promotes an elevation of *NEUROD1*. However, while this WNT-signalling-dependent transcription factor is essential for neural progenitor survival and neurogenesis (Kuwabara et al., 2009), it is also required for ERK-regulated pro-neural activity (Lee et al., 2020); indicating multiple levels of regulatory control. Consistent with our work, both *NeuroD1* and *Msx2* can be regulated by β-catenin-dependent WNT signalling (Kuwabara et al., 2009), and the marked decrease in expression of *WNT5A* in response to the removal of TGFβ3 and dbcAMP may be consistent with increased β-catenin-dependent signalling in these cultures, as proposed previously (Haynes et al., 2021).

While DAPT is routinely included in cultures to encourage differentiation, dbcAMP to increase TH expression (sometimes taken for promoting survival) and TGFβ3 to improve survival, the ability of these three factors to regulate markers of an immature phenotype; *MSX2, NEUROD1* and *nestin* indicate that their removal, even at late stages of culture, has profound effects upon culture phenotype. Perhaps most significantly, with respect to TGFβ3 and DAPT, removal of these factors may lead to increased glial commitment while possibly also contributing to other markers of phenotype specification.

#### Early markers of an mDA phenotype: OTX2, EN1/2 FOXA2, LMX1A

Generally, the floor plate markers *LMX1A, FOXA2, EN1/2* and *OTX2* (Nakatani et al., 2010; Aguila et al., 2014; Arenas et al., 2015) were robustly expressed throughout differentiation up until termination at ∼ day 65. Overall, *LMX1A* was the least impacted transcription factor as it was unaffected by any culture manipulation. We have, however, already shown *LMX1A* expression to be negatively influenced by the removal of β-catenin signalling (Haynes et al., 2021) which is broadly consistent with the idea that endogenous WNT, or at least β-catenin signalling networks are intact and functional in these cultures. *FOXA2* was, however, positively under the influence of TGFβ3 since its removal promoted a loss of transcript, a finding consistent with SMAD-dependent changes in Foxa2 (Cai et al., 2013). This downregulation of *FOXA2* was anticipated as previous studies have shown TGFβ signaling regulates the expression lncRNA DEANR1 (Definitive Endoderm Associated long Non-coding RNA1) by positively regulating endoderm factor FOXA2 expression (Mullen and Wrana, 2017).

In contrast to *FOXA2*, the engrailed genes (*EN1* and *EN2*) were unaffected by the removal of TGFβ3 signalling but greatly impacted by the loss of dbcAMP. These genes are key players in mDA neuron patterning, axonal guidance, neuron specification and maintenance (Alves dos Santos and Smidt, 2011) (Danielian and McMahon, 1996). These genes are generally considered as WNT-dependent transcription factors (Danielian and McMahon, 1996) and are expressed in multiple dopaminergic neuronal subtypes (La Manno et al., 2016). There is, however, significant overlap between WNT and protein kinase A signalling since PKA phosphorylation of β-catenin at Ser^522^, a canonical WNT signalling independent phosphorylation site (Luckert et al., 2011) promotes β-catenin accumulation (Cognard et al., 2013). En1 (along with Wnt1) expression has been shown to be dependent upon Ras activation (Vennemann et al., 2008), a finding supported by the direct induction of En1 expression by FGF8 (Harada et al., 2016), *EN1* expression can also be induced by TGFβ in a SMAD3-dependent manner (Gyorfi et al., 2021). Although TGFβ3 had no effect upon *EN1* expression in our cultures, protein kinase A can facilitate ERK signalling through phosphorylation of the scaffold protein, KSR-1 (Smith et al., 2012), indicating a potential for TGFβ3 pathway-dependent expression to be modulated by PKA (i.e., dbcAMP). That *EN2* could be regulated only by the removal of dbcAMP may indicate that similar mechanisms to those regulating *EN1* are at play. However, one major point of difference is that *EN1*, but not *EN2*, is also downregulated by the removal of DAPT and presumably the activation of notch signalling. While this may be consistent with the differences in putative transcription factor binding site regulation of EN1 and EN2 expression, how this occurs is not immediately clear.

Lastly, *OTX2* expression was downregulated by the removal of DAPT and TGFβ3. OTX2 can be driven by both notch signalling (Dvoriantchikova et al., 2015) and also by MAPK (Hongo and Okamoto, 2022). Moreover, a recent study reported reduced OTX2 expression in iPSC-derived spheroids incubated with WNT and DAPT (CHIR + DAPT) (Bejoy et al., 2020). While we observed no change in the expression of WNT1, previous literature reported that the Notch modulator *Lfng* and the Notch ligand *Ser1* are also expressed in a compartment-restricted fashion at the midbrain-hindbrain boundary (MHB), and have a function in stabilization of the boundary and in the regulation of expression of the organizer genes *Fgf8* and *Wnt1* (Tossell et al., 2011)*.* Whether these pathways act independently or interact to control expression is unknown.

These studies indicate that cultures maintain the expression of early markers of neural and floorplate phenotypes. While these markers are generally thought of as early factors in differentiation programs, they do persist late in cultures, and their expression patterns are modified by the removal of a variety of factors which broadly impacts the overall phenotype of cultures.

#### Late markers of an mDA phenotype: SOX6, NR4A2, PITX3, TH, transporters, KCNJ6, ALDH1A1, and CALB1

Even though SOX6 appears early in development it is often thought of for its association with the A9 subset of dopaminergic neurons (OTX2 is often considered in association with A10 neurons) (Panman et al., 2014). That SOX6 expression increases after the removal of TGFβ3 is somewhat at odds with the idea that *SOX6* is an important downstream mediator of BMP-2 signalling in chondrogenesis (Fernández-Lloris et al., 2003), although we have previously shown *SOX6* expression to be regulated by WNT signalling (albeit in the presence of TGFβ3, dbcAMP and DAPT) (Haynes et al., 2021).

PITX3 and NR4A2 are more typically expressed later in differentiation and in both A9 and A10 neurons (Mesman and Smidt, 2020). There is evidence of a great deal of interplay between PITX3, EN1 and NR4A2. *Pitx3* has been proposed as a regulator of mDA (Saucedo-Cardenas et al., 1998; Lebel et al., 2001) and may cooperate with *Nr4a2* to promote survival and terminal differentiation (Nunes et al., 2003; Jacobs et al., 2009; Chakrabarty et al., 2012), although other studies have also shown that *Nr4a2* knockout has no influence on *Pitx3* expression, suggesting independence of the two pathways (Zetterstrom et al., 1997; Castillo et al., 1998; Saucedo-Cardenas et al., 1998). Consistent with the idea of pathway independence we saw no reduction of *NR4A2* expression when *PITX3* was reduced following the removal of dbcAMP. Thus, while NR4A2 is thought to be necessary in establishing a Th-positive dopaminergic phenotype (Zetterstrom et al., 1997; Saucedo-Cardenas et al., 1998) our evidence supports the idea that PITX3 and NR4A2 expression occur independently; at least with respect to effects of dbcAMP. En1^−/−^ mice embryos show (rostrolateral but not mediocaudal) reductions in Pitx3, Th and Slc6a3, but no changes in Nr4a2; Veenvliet and others speculated that En1 and Pitx3 cooperatively drive expression of Nr4a2 target genes *Slc6a3* and *Th* in a rostrolateral subset of mdDA neurons but where Pitx3 represses some En1-driven genes (Veenvliet et al., 2013). In the absence of dbcAMP, we saw reductions in *EN1*, *PITX3* and *TH*, but not *SLC6A3* or *NR4A2*. Interestingly, the removal of DAPT led to a reduction in *EN1* and *KCNJ6* expression but not *TH* or *PITX3*, possibly indicating what may be construed as a loss of the A9, but not dopaminergic neuron phenotype. This might be consistent with an increase in the A10 marker, *OTX2*, but it is not consistent with the lack of change in expression of the other VTA marker, *CALB1* indicating distinct regulatory mechanisms.

The effects of TGFβ3 and dbcAMP upon TH expression are not surprising as a substantial body of evidence shows the impact modulating cAMP and TGFβ has on cultures. For example, dbcAMP accelerates neuron maturation (Lepski et al., 2013), promotes dendritic arborization and increases density of high-affinity dopamine uptake sites and choline acetyltransferase activity (Mena et al., 1995). Cyclic AMP is a powerful inducer of TH gene transcription *via* transcription factors such as CREB and ATF1, which, in conjunction with the coactivator cyclic AMP response element-binding protein (CBP)/p300 control transcriptional regulation of the *TH* gene (Mena et al., 1995; Lim et al., 2000). A recent study reported that increasing cAMP levels by inhibiting phosphodiesterase activity is sufficient to support the rapid functional maturation of neuronal progenitors into fully functional neurons (Lepski et al., 2013). In our study, the removal of dbcAMP from cultures relatively late in maturation produced profound changes phenotype, without necessarily promoting much change in neuronal phenotype (although we saw a small reduction in *TUBB3* expression). Similarly, our finding that mDA phenotype was compromised by the loss of TGFβ3 is consistent with data showing that blocking BMP signalling results in a loss of TH-positive mDA neurons (Jovanovic et al., 2018), as well as data showing that Tgfβ2−/− and Tgfβ3−/− mice have a severe reduction in mDA neuron numbers (Airaksinen and Saarma, 2002). Transforming growth factor-βs are widely recognized as prototypical multifunctional growth factors and master switches that regulate key events in development, disease, and repair (Krieglstein et al., 2002). BMP and TGFβ pathway mediators are critical upstream regulators of Wnt signalling during midbrain dopaminergic differentiation (Cai et al., 2013). TGFβ directly increases the number of Nr4a2-and Th- immunoreactive cells, which can be further increased when cultures are incubated with TGFβ in combination with Shh and fibroblast growth factor 8 (FGF8) (Airaksinen and Saarma, 2002). TGFβ3 may also ectopically induce TH-immunopositive cells in dorsal mesencephalon *in vitro*, in a Shh- and FGF8-independent manner (Roussa et al., 2006), a finding consistent with our data showing that the removal of TGFβ3 from our cultures impacted *TH* expression, albeit without effect upon *NR4A2*. There is evidence that the effect of TGFβ3 in maintaining *TH* expression in mature neurons may be *via* an indirect effect upon BDNF and GDNF; neurotrophic factors that belong to the TGFβ superfamily (Airaksinen and Saarma, 2002; Binder and Scharfman, 2004). Since GDNF and BDNF are still present in our cultures this remains a possibility.

WNT5A expression was greatly impacted by the removal of dbcAMP or TGFβ3. WNT5A is predominantly a non-β-catenin-dependent signalling member of the WNT family, which regulates a number of essential processes in early development (Topol et al., 2003). In mice, *Wnt5a* predominantly increases differentiation of *Nr4a2* precursors into mDA neurons (Castelo-Branco et al., 2003), while *Wnt5a* deletion increases progenitor proliferation *via* activation of the GTPase, Rac1 (Andersson et al., 2008). Moreover, *WNT5A* regulates mDA axon growth and guidance both *in vitro* and *in vivo* (Blakely et al., 2011) and improves the differentiation and functional integration of stem cell-derived DA neurons *in vivo* (Parish et al., 2008). In our hands, a significant loss of *WNT5A* but not *WNT1* was also observed in the absence of either dbcAMP or TGFβ3. There is clear evidence of crosstalk between β-catenin dependent WNT and cAMP/PKA signalling pathways (Hino et al., 2005; Taurin et al., 2006). Protein kinase A (PKA) phosphorylates β -catenin, promoting binding to its transcriptional coactivator, CREB-binding protein (Taurin et al., 2006). Furthermore, cAMP activation by parathyroid hormone (PTH) and ATP also inhibits the destruction complex, thereby reducing β-catenin degradation and promoting transcriptional activity (Taurin et al., 2006). Both pathways are known to stimulate cell proliferation (Takahashi et al., 2004; Clevers et al., 2014; Li et al., 2018). Similarly, TGFβ pathway mediators are critical upstream regulators of Wnt1-Lmx1a signalling in mDA progenitors (Cai et al., 2013) (Anderegg et al., 2013) (Claude et al., 2019). However, TGFβ directly regulates the expression of Wnt5a in the mammary gland (Serra et al., 2011) and Wnt5a potentiates TGFβ Signaling to promote colonic crypt regeneration after tissue injury in the mammalian intestinal epithelium (Miyoshi et al., 2012). Thus the interactions between cAMP, TGFβ, and WNT signalling systems; where cAMP-dependent suppression of TGFβ signalling (Jia et al., 2009) as well as a loss of Wnt5a or TGFβ resulting in a stabilization of β-catenin (Jia et al., 2009; Estarás et al., 2012) confound interpretation of this data. In the absence of dbcAMP, we found a profound loss of WNT5A, which supports the idea that WNT signalling is compromised and leads to changes in mDA phenotype even though our WNT5A immunolabelling revealed that WNT5A was abundantly expressed in most cell types, not just LMX1A-eGFP positive cells. These studies highlight the problems with ascribing activities to the removal of particular factors in as much as the resultant effects may be mediated *via* third-party signalling systems.

In conclusion, our findings demonstrate that common media additives are critical to maintaining the appearance of a widespread mDA neuron phenotype. These additives selectively affect many of the genes associated with mDA progenitor and mature neuron phenotype (NES, NEUROD1, FOXA2, SOX6, KCNJ6, PITX3, EN1/2, OTX2 and TH) as well as astroglial differentiation and activation (GFAP and S100B). Generally, the removal of any one of dbcAMP, TGFβ3 or DAPT is likely to give the impression of a loss of some aspect of an A9 or mDA phenotype from cultures. That dbcAMP and TGFβ3 regulated WNT5A expression indicates a dynamic endogenous WNT signalling network present in cultures. Consistent with this idea we observed a considerable lack of consistency between the putative transcription factors regulating expression of genes (Supplementary Table S5) and the impacts that removal of the activators of these signalling systems (ie. dbcAMP, TGFβ3 and DAPT) have upon gene expression. Understanding that these media supplements serve to elevate the expression of mDA (while suppressing astroglial) markers is of great significance to understand the outcomes of progenitor transplantation for Parkinson’s Disease. Thus, our cultures appear quite plastic with respect to the expression of markers of a dopaminergic neuronal phenotype, and we would argue that much of this culture-wide expression is dependent upon the addition of exogenous additives as well as the regulation of endogenous signalling systems (especially WNTS). We believe that this raises an important issue about the success of transplantation therapies where, for example the transplanted progenitors are under the influence of brain chemistry rather than exogenously applied factors designed to promote TH expression. Similarly, the influence of these factors is also likely to permeate into *in vitro* studies of survival and function.

## Data Availability

The raw data supporting the conclusions of this article will be made available by the authors, without undue reservation.
