# Supplementary material for: TGFβ3, dibutyryl cAMP and a notch inhibitor modulate phenotype late in stem cell-derived dopaminergic neuron maturation
Source: Front Cell Dev Biol. 2023 Feb 1;11:1111705. doi: 10.3389/fcell.2023.1111705 (PMC9928866; doi:10.3389/fcell.2023.1111705)
Supplement: Supplementary file 1 [file Table1.DOCX]

Supplementary Material

SUPPLEMENTAL INFORMATION

### S1 Table Primary and secondary antibodies used in this study.

| Antibody | Supplier | Catalogue number |
| --- | --- | --- |
| Anti-beta III Tubulin (chicken) | Abcam | ab41489 |
| Anti-Tyrosine Hydroxylase (sheep) | Abcam | ab113 |
| Anti-Map2 (chicken) | Abcam | ab5392 |
| Anti-PITX3 (rabbit) | ThermoFisher | 38-2850 |
| Anti-GIRK2/KCNJ6 (goat) | Abcam | Ab65096 |
| Anti-WNT5A (rabbit) | Abcam | ab179824 |
| Anti-GFAP antibody (goat) | Abcam | ab53554 |
| Donkey Anti-Rabbit IgG H&L (Alexa Fluor® 488) | Abcam | ab150073 |
| Donkey Anti Sheep IgG HL Alexa Fluor 555 | Abcam | ab150178 |
| Donkey Anti-Rabbit IgG H&L (Alexa Fluor® 555) | Abcam | Ab150062 |
| Donkey Anti-Chicken IgY 647 | Jackson | 703-605-155 |

### S2 Taqman probes used for quantitative PCR.

| **Taqman probe** | **Gene expression ID** |
| --- | --- |
| ALDH1A1 | Hs00946916_m1 |
| CALB1 | Hs01077197_m1 |
| FOXA2 | Hs00232764_m1 |
| GFAP | Hs00909233_m1 |
| KCNJ6 | Hs01040524_m1 |
| LMX1A | Hs00892663_m1 |
| MSX2 | Hs00751239_s1 |
| NES | Hs04187831_g1 |
| NEUROD1 | Hs01922995_s1 |
| NR4A2 | Hs01118813_m1 |
| OTX2 | Hs00222238_m1 |
| PITX3 | Hs00374504_m1 |
| S100B | Hs00902901_m1 |
| SOX6 | Hs00264525_m1 |
| TH | Hs00165941_m1 |
| TUBB3 | Hs00801390_s1 |
| SLC18A2 | Hs00997364_m1 |
| SLC6A3 | Hs00997364_m1 |
| WNT1 | Hs01011247_m1 |
| WNT5A | Hs00998537_m1 |
| HPRT1 | Hs02800695_m1 |
| TBP1 | Hs00427620_m1 |
| NR0B1 | Hs03043658_m1 |
| NR0B2 | Hs00222677_m1 |

**S3 Table PCR Data**

| -dbcAMP | LMX1A | MSX2 | EN1 | EN2 | FOXA2 | NR4A2 | PITX3 | TH | SLC6A3 | SLC18A2 | KCJN6 |
| --- | --- | --- | --- | --- | --- | --- | --- | --- | --- | --- | --- |
|  | 1.311 | 8.664 | 0.223 | 0.142 | 1.536 | 0.888 | 0.166 | 0.430 | 1.530 | 3.038 | 0.812 |
|  | 0.699 | 11.547 | 0.189 | 1.124 | 0.834 | 2.069 | 0.212 | 0.343 | 0.876 | 2.032 | 1.238 |
|  | 1.760 | 13.232 | 0.074 | 0.144 | 1.584 | 1.588 | 0.132 | 0.558 | 1.461 | 1.629 | 0.950 |
|  | 0.711 |  | 0.088 | 0.089 |  | 0.518 | 0.679 | 0.625 | 0.574 |  |  |
|  |  |  | 0.303 | 0.534 |  | 0.355 | 0.471 | 0.336 |  |  |  |
|  |  |  |  |  |  | 0.710 | 0.774 | 0.795 |  |  |  |
|  |  |  |  |  |  | 0.661 | 0.659 | 0.452 |  |  |  |
|  |  |  |  |  |  |  |  |  |  |  |  |
|  | ALDH1A1 | CALB1 | SOX6 | OTX2 | GFAP | S100B | NES | TUBB3 | NEUROD1 | WNT1 | WNT5A |
|  | 0.601 | 1.204 | 0.656 | 1.344 | 3.294 | 1.567 | 0.147 | 0.915 | 4.060 | 0.199 | 0.266 |
|  | 0.739 | 1.782 | 0.610 | 0.793 | 9.452 | 0.815 | 0.115 | 0.740 | 17.004 | 0.225 | 0.251 |
|  | 0.468 | 1.296 | 1.522 | 3.357 | 21.127 | 1.364 | 0.239 | 0.869 | 6.965 | 1.343 | 0.271 |
|  |  |  |  |  | 1.554 |  | 2.306 | 0.943 |  |  |  |
|  |  |  |  |  | 2.957 |  | 0.929 | 0.723 |  |  |  |

| -TGFβ3 | LMX1A | MSX2 | EN1 | EN2 | FOXA2 | NR4A2 | PITX3 | TH | SLC6A3 | SLC18A2 | KCNJ6 |
| --- | --- | --- | --- | --- | --- | --- | --- | --- | --- | --- | --- |
|  | 0.333 | 9.208 | 0.759 | 1.543 | 0.562 | 0.590 | 0.697 | 0.184 | 0.686 | 0.525 | 0.084 |
|  | 0.635 | 5.951 | 0.074 | 0.717 | 0.501 | 2.213 | 0.774 | 0.339 | 0.459 | 0.379 | 0.362 |
|  | 0.878 | 13.942 | 0.673 | 1.538 | 0.500 | 1.056 | 1.079 | 0.377 | 0.291 | 1.790 | 0.111 |
|  |  |  |  |  |  |  |  |  |  |  |  |
|  | ALDH1A1 | CALB1 | SOX6 | OTX2 | GFAP | S100B | NES | TUBB3 | NEUROD1 | WNT1 | WNT5A |
|  | 0.436 | 1.907 | 2.277 | 3.892 | 1.284 | 4.775 | 0.163 | 1.116 | 5.113 | 2.822 | 0.455 |
|  | 0.380 | 1.678 | 2.927 | 3.251 | 5.744 | 10.605 | 0.208 | 0.669 | 14.050 | 5.399 | 0.348 |
|  | 0.806 | 0.907 | 2.224 | 6.769 | 9.524 | 3.030 | 0.301 | 0.636 | 6.431 | 0.751 | 0.397 |

| -DAPT | LMX1A | MSX2 | EN1 | EN2 | FOXA2 | NR4A2 | PITX3 | TH | SLC6A3 | SLC18A2 | KCNJ6 |
| --- | --- | --- | --- | --- | --- | --- | --- | --- | --- | --- | --- |
|  | 0.803 | 8.204 | 0.213 | 1.628 | 0.601 | 0.884 | 0.506 | 0.479 | 0.943 | 1.141 | 0.448 |
|  | 1.044 | 11.942 | 0.333 | 1.614 | 0.197 | 1.922 | 0.331 | 0.837 | 0.649 | 0.824 | 0.304 |
|  | 0.618 | 19.220 | 0.193 | 1.208 | 0.338 | 1.444 | 0.694 | 0.872 | 0.444 | 0.447 | 0.196 |
|  |  |  |  |  |  |  |  |  |  |  |  |
|  | ALDH1A1 | CALB1 | SOX6 | OTX2 | GFAP | S100B | NES | TUBB3 | NEUROD1 | WNT1 | WNT5A |
|  | 0.653 | 0.929 | 0.807 | 2.623 | 3.929 | 5.911 | 0.203 | 0.522 | 0.942 | 2.615 | 0.908 |
|  | 0.792 | 0.942 | 1.342 | 3.379 | 8.766 | 4.208 | 0.100 | 1.211 | 5.653 | 0.289 | 0.673 |
|  | 0.555 | 0.768 | 1.167 | 6.644 | 15.542 | 6.640 | 0.115 | 0.919 | 2.754 | 6.678 | 1.048 |

**S4 Table Thresholding Data**

| GFAP |  | Area GFAP | Area DAPI | Min Thr GFAP | Min Thr DAPI | Max Thr | Proportion | %DAPI |
| --- | --- | --- | --- | --- | --- | --- | --- | --- |
|  | Control | 69833.794 | 283867.791 | 600 | 1650 | 65535 | 0.246008164 | 24.60081637 |
|  |  | 53246.922 | 284424.808 | 600 | 1650 | 65535 | 0.187209134 | 18.7209134 |
|  |  | 65978.806 | 309816.667 | 600 | 1650 | 65535 | 0.212960802 | 21.29608024 |
|  | DAPT | 119565.493 | 160336.591 | 600 | 1650 | 65535 | 0.745715574 | 74.57155741 |
|  |  | 112217.731 | 168096.789 | 600 | 1650 | 65535 | 0.667578076 | 66.75780761 |
|  |  | 96500.248 | 172007.327 | 600 | 1650 | 65535 | 0.561024055 | 56.10240545 |
|  | dbcAMP | 160771.856 | 256061.048 | 600 | 1650 | 65535 | 0.627865336 | 62.78653362 |
|  |  | 157680.865 | 184957.985 | 600 | 1650 | 65535 | 0.852522615 | 85.25226148 |
|  |  | 231376.876 | 270377.236 | 600 | 1650 | 65535 | 0.855755756 | 85.5755756 |
|  | TGFb3 | 106217.617 | 315577.461 | 600 | 1650 | 65535 | 0.336581759 | 33.65817592 |
|  |  | 127481.687 | 288127.225 | 600 | 1650 | 65535 | 0.442449293 | 44.2449293 |
|  |  | 68209.92 | 362254.315 | 600 | 1650 | 65535 | 0.188292912 | 18.82929124 |

| KCNJ6 |  | Area KCNJ6 | Area DAPI | Min Thr KCNJ6 | Min Thr DAPI | Max Thr | Proportion | %DAPI |
| --- | --- | --- | --- | --- | --- | --- | --- | --- |
|  | Control | 118980.32 | 336555.412 | 500 | 1800 | 65535 | 0.353523716 | 35.35237163 |
|  |  | 63648.007 | 109338.286 | 500 | 1800 | 65535 | 0.582120036 | 58.21200362 |
|  |  | 137798.306 | 334688.033 | 500 | 1800 | 65535 | 0.41172164 | 41.17216405 |
|  |  | 71171.168 | 137666.281 | 500 | 1800 | 65535 | 0.516983298 | 51.69832982 |
|  | DAPT | 85201.238 | 280454.156 | 500 | 1800 | 65535 | 0.303797381 | 30.37973807 |
|  |  | 95873.604 | 301817.149 | 500 | 1800 | 65535 | 0.317654594 | 31.76545942 |
|  |  | 101080.044 | 307498.804 | 500 | 1800 | 65535 | 0.328716869 | 32.87168688 |
|  |  | 23796.14 | 76898.861 | 500 | 1800 | 65535 | 0.309447236 | 30.94472362 |
|  | dbcAMP | 73395.814 | 294425.632 | 500 | 1800 | 65535 | 0.249284729 | 24.92847294 |
|  |  | 100161.573 | 214662.164 | 500 | 1800 | 65535 | 0.466600966 | 46.66009656 |
|  |  | 100930.897 | 326608.615 | 500 | 1800 | 65535 | 0.309027051 | 30.90270506 |
|  |  | 53331.387 | 187372.489 | 500 | 1800 | 65535 | 0.284627627 | 28.46276275 |
|  | TGFb3 | 82247.98 | 288966.556 | 500 | 1800 | 65535 | 0.284628025 | 28.46280246 |
|  |  | 90199.558 | 276087.047 | 500 | 1800 | 65535 | 0.326706953 | 32.67069534 |
|  |  | 106888.397 | 318242.699 | 500 | 1800 | 65535 | 0.335870697 | 33.58706966 |
|  |  | 31764.839 | 124222.525 | 500 | 1800 | 65535 | 0.255709172 | 25.57091719 |

| WNT5A |  | Area WNT5A | Area DAPI | Area LMX1A | Min Thr WNT5A | Min Thr DAPI | Max Thr | Proportion | %DAPI |
| --- | --- | --- | --- | --- | --- | --- | --- | --- | --- |
|  | Control | 209910.774 | 295832.634 | 68471.307 | 700 | 1600 | 65535 | 0.709559223 | 70.95592 |
|  |  | 227318.333 | 315212.584 | 37654.615 | 700 | 1600 | 65535 | 0.721158813 | 72.11588 |
|  |  | 126151.921 | 274728.365 | 149103.404 | 700 | 1600 | 65535 | 0.459187827 | 45.91878 |
|  | dbcAMP | 67615.996 | 312499.787 | 100627.657 | 700 | 1600 | 65535 | 0.216371335 | 21.63713 |
|  |  | 79171.447 | 315212.584 | 103743.379 | 700 | 1600 | 65535 | 0.251168421 | 25.11684 |
|  |  | 45065.536 | 172758.007 | 57472.874 | 700 | 1600 | 65535 | 0.260859319 | 26.08593 |
|  | TGFb3 | 43535.64 | 156110.259 | 12476.585 | 700 | 1600 | 65535 | 0.278877508 | 27.88775 |
|  |  | 42867.143 | 139186.665 | 66723.018 | 700 | 1600 | 65535 | 0.307983117 | 30.79831 |
|  |  | 85340.112 | 263123.072 | 116261.816 | 700 | 1600 | 65535 | 0.324335344 | 32.43353 |
|  | DAPT | 72002.51 | 170980.421 | 34650.754 | 700 | 1600 | 65535 | 0.421115526 | 42.11155 |
|  |  | 42530.041 | 116383.949 | 36585.857 | 700 | 1600 | 65535 | 0.36542875 | 36.54287 |
|  |  | 56788.016 | 152900.939 | 48473.083 | 700 | 1600 | 65535 | 0.371403972 | 37.1404 |

| LMX1A |  | Area LMX1A | Area DAPI | Min Thr LMX1A | Min Thr DAPI | LMX1A/DAPI | %L |
| --- | --- | --- | --- | --- | --- | --- | --- |
|  | Control | 98308.653 | 336555.412 | 1500 | 1600 | 0.292102428 | 29.2102428 |
|  |  | 60707.685 | 109338.286 | 1500 | 1600 | 0.555228065 | 55.52280653 |
|  |  | 74548.658 | 334688.033 | 1500 | 1600 | 0.222740734 | 22.27407336 |
|  |  | 68471.307 | 295832.634 | 1500 | 1600 | 0.231452852 | 23.14528525 |
|  |  | 37654.615 | 315212.584 | 1500 | 1600 | 0.119457842 | 11.94578418 |
|  |  | 149103.404 | 274728.365 | 1500 | 1600 | 0.542730286 | 54.27302856 |
|  | DAPT | 50053.583 | 280454.156 | 1500 | 1600 | 0.178473315 | 17.84733153 |
|  |  | 65304.982 | 301817.149 | 1500 | 1600 | 0.216372669 | 21.63726687 |
|  |  | 113462.27 | 307498.804 | 1500 | 1600 | 0.368984427 | 36.8984427 |
|  |  | 34650.754 | 170980.421 | 1500 | 1600 | 0.202659192 | 20.26591922 |
|  |  | 36585.857 | 116383.949 | 1500 | 1600 | 0.314354834 | 31.43548343 |
|  |  | 48473.083 | 152900.939 | 1500 | 1600 | 0.317022795 | 31.70227947 |
|  | dbcAMP | 60951.19 | 294425.632 | 1500 | 1600 | 0.207017268 | 20.70172681 |
|  |  | 88954.258 | 214662.164 | 1500 | 1600 | 0.414391881 | 41.43918814 |
|  |  | 86975.781 | 326608.615 | 1500 | 1600 | 0.266299715 | 26.62997147 |
|  |  | 100627.657 | 312499.787 | 1500 | 1600 | 0.322008722 | 32.20087219 |
|  |  | 103743.379 | 315212.584 | 1500 | 1600 | 0.329121946 | 32.91219458 |
|  |  | 57472.874 | 172758.007 | 1500 | 1600 | 0.332678496 | 33.26784963 |
|  | TGFb3 | 65747.096 | 288966.556 | 1500 | 1600 | 0.227524932 | 22.7524932 |
|  |  | 62189.641 | 276087.047 | 1500 | 1600 | 0.225253744 | 22.5253744 |
|  |  | 91567.751 | 318242.699 | 1500 | 1600 | 0.287729306 | 28.77293062 |
|  |  | 12476.585 | 156110.259 | 1500 | 1600 | 0.079921621 | 7.992162129 |
|  |  | 66723.018 | 139186.665 | 1500 | 1600 | 0.479377949 | 47.9377949 |
|  |  | 116261.816 | 263123.072 | 1500 | 1600 | 0.44185337 | 44.18533697 |

### S5 Table MotifMap analysis of putative transcriptional control.

# Putative transcription factor binding sites regulating expression of genes of interest (GOI).

Sites were estimated using MotifMap. GOI in bold, putative number of binding sites are to the right. LEF1, HNF4 and NEURO D sites are colored for ease of identification.

Note: Putative binding sites are not corrected for overlap or orientation

| ALDH1A1 | | | | CALB1 | | | | SLC18A2 | | |  | SLC6A3 | | EN1 | | | | | |
| --- | --- | --- | --- | --- | --- | --- | --- | --- | --- | --- | --- | --- | --- | --- | --- | --- | --- | --- | --- |
| Neuro D | 10 | HNF4 | 1 | IRF-1 | 6 | E2A | 1 | MZF1_4 | 15 | AML1 | 1 | MZF1_1-4 | 2 | MZF1 | 21 | Lhx3 | 2 | HIF-2alpha | 1 |
| MZF1_4 | 5 | IPF1 | 1 | LEF1 | 6 | E47 | 1 | CREB | 14 | AP-2rep | 1 | ZNF354C | 2 | ZNF354C | 12 | Lhx5 | 2 | HMG IY | 1 |
| NKX3-1 | 5 | NURR1 | 1 | IPF1 | 5 | HEB | 1 | HNF4 | 13 | CRX | 1 | DBP | 1 | HNF4 | 10 | Lmx-1 | 2 | HOXA4 | 1 |
| CRX | 4 | PU.1 | 1 | MZF1_1-4 | 5 | HOXC-8 | 1 | Neuro D | 8 | E2A | 1 | E2A | 1 | STAT1 | 10 | MAFA | 2 | HOXA7 | 1 |
| SPI-B | 4 | SOX4 | 1 | TEF-1 | 5 | HOXC10 | 1 | ZNF354C | 8 | ETS1 | 1 | HNF4 | 1 | GATA | 9 | MIZF | 2 | HOXB9 | 1 |
| LBP-1 | 3 | STATx | 1 | CRX | 4 | HOXC11 | 1 | LEF1 | 7 | GABP-alpha | 1 | LEF1 | 1 | ETS1 | 6 | Myc | 2 | HOXC4 | 1 |
| LEF1 | 3 | TAL1::TCF3 | 1 | CTCF | 4 | HOXC12 | 1 | YY1 | 7 | HMG IY | 1 | STAT1 | 1 | Neuro D | 6 | NFAT2 | 2 | IRF8 | 1 |
| DBP | 2 | TBP | 1 | GLI1 | 4 | MAFA | 1 | Nkx3-2 | 6 | HOXA7 | 1 | TEF-1 | 1 | TEF-1 | 6 | Nkx3-2 | 2 | K-2b | 1 |
| E4BP4 | 2 |  |  | HMG IY | 4 | Myf | 1 | TEF-1 | 6 | IRF2 | 1 |  |  | USF | 6 | RBP-Jkappa | 2 | NERF1a | 1 |
| FOXM1 | 2 |  |  | Ets | 3 | MyoD | 1 | STAT3 | 5 | IRF8 | 1 |  |  | LEF1 | 5 | TRF1 | 2 | NF-muE1 | 1 |
| HMG IY | 2 |  |  | GATA | 3 | NFIL3 | 1 | ATF | 4 | Lyf-1 | 1 |  |  | SOX10 | 5 | AP-3 | 1 | Nkx2-5 | 1 |
| IRF-1 | 2 |  |  | MAFB | 3 | NKX3-1 | 1 | ATF1 | 3 | Myc | 1 |  |  | IRF-1 | 4 | AREB6 | 1 | NUR77 | 1 |
| IRF8 | 2 |  |  | MEF-2 | 3 | Nkx3-2 | 1 | GATA2 | 3 | NURR1 | 1 |  |  | MAFB | 4 | ATF6 | 1 | OCT01 | 1 |
| NFIL3 | 2 |  |  | OCT1 | 3 | OCT1 | 1 | ATF2:c-Jun | 2 | Pax-2 | 1 |  |  | NURR1 | 4 | BEN | 1 | OCT-x | 1 |
| RFX1 | 2 |  |  | STAT5A | 3 | POU2F3 | 1 | ATF3 | 2 | Pax-4 | 1 |  |  | YY1 | 4 | BRCA1 | 1 | Octamer | 1 |
| TEF-1 | 2 |  |  | Brachyury | 2 | PXR (PXR:RXR) | 1 | IPF1 | 2 | Prop-1 | 1 |  |  | En-1 | 3 | Brn-4 | 1 | Pit-1 | 1 |
| YY1 | 2 |  |  | HOXA7 | 2 | RXRA::VDR | 1 | LBP-1 | 2 | RORBETA | 1 |  |  | GABP-alpha | 3 | CART1 | 1 | PMX2B | 1 |
| ZNF354C | 2 |  |  | NFAT2 | 2 | SPI1 | 1 | MAFB | 2 | SPIB | 1 |  |  | MyoD | 3 | CREB | 1 | Prop-1 | 1 |
| AP-3 | 1 |  |  | NURR1 | 2 | TAL1 | 1 | PUR1 | 2 | TAL1 | 1 |  |  | Pax-2 | 3 | CTCF | 1 | Shox2 | 1 |
| E2A | 1 |  |  | SPIB | 2 | TBX | 1 | RORA_1 | 2 |  |  |  |  | AP-2rep | 2 | E12 | 1 | SPIB | 1 |
| E47 | 1 |  |  | TBP | 2 | TCF-4 | 1 | RORalpha | 2 |  |  |  |  | CDP | 2 | E2F1 | 1 | ZBRK1 | 1 |
| Ets | 1 |  |  | ZNF354C | 2 | Tel-2 | 1 | SOX10 | 2 |  |  |  |  | CRX | 2 | E47 | 1 |  |  |
| ETS2 | 1 |  |  | AP-3 | 1 | VDR:RXR | 1 | SPI1 | 2 |  |  |  |  | DBP | 2 | E4F1 | 1 |  |  |
| FXR | 1 |  |  | c-Ets-1 | 1 | YY1 | 1 | USF2 | 2 |  |  |  |  | HSF | 2 | Ebox | 1 |  |  |
| GR | 1 |  |  | CREB | 1 |  |  | Vax-2 | 2 |  |  |  |  | LBP-1 | 2 | HEB | 1 |  |  |

| EN2 | | | | FOXA2 | | GFAP | | KCNJ6 | | | | LMX1A | | MAP2 | |
| --- | --- | --- | --- | --- | --- | --- | --- | --- | --- | --- | --- | --- | --- | --- | --- |
| MZF1_1-4 | 17 | E2A | 2 | MZF1_1-4 | 7 | MZF1 | 8 | GATA | 14 | Ikaros | 1 | HOXA4 | 6 | MZF1_1-4 | 9 |
| Neuro D | 14 | HMG IY | 2 | HNF4 | 4 | HNF4 | 6 | NKX3-1 | 8 | IPF1 | 1 | CART1 | 4 | Neuro D | 6 |
| ZNF354C | 14 | LEF1 | 2 | TBX | 4 | Neuro D | 4 | LEF1 | 7 | LBP-1 | 1 | ETS1 | 4 | HNF4 | 4 |
| USF | 11 | MAFA | 2 | ZNF354C | 3 | TEF | 3 | ZNF354C | 7 | MAFA | 1 | GABP-alpha | 4 | STAT3 | 4 |
| CREB | 9 | NF-Y | 2 | Brachyury | 2 | BRCA1 | 2 | MZF1_1-4 | 6 | Myc | 1 | GATA2 | 3 | GATA3 | 3 |
| ETS2 | 8 | Otx1 | 2 | E12 | 2 | HOXA7 | 2 | SOX10 | 6 | NFAT2 | 1 | HNF4 | 3 | HMG IY | 3 |
| SPIB | 8 | PU.1 | 2 | GLI2 | 2 | Nkx2-5 | 2 | HNF4 | 4 | NFIL3 | 1 | MZF1_1-4 | 3 | MAFB | 3 |
| ETS1 | 7 | AP-3 | 1 | SOX10 | 2 | ETS2 | 1 | IRF-1 | 4 | TBX5 | 1 | Alx-4 | 2 | ETS1 | 2 |
| GABP-alpha | 7 | BEN | 1 | ATF6 | 1 | GATA2 | 1 | CRX | 3 | YY1 | 1 | CREB | 2 | GABP-alpha | 2 |
| TEF-1 | 7 | deltaEF1 | 1 | c-Myc:Max | 1 | LBP-1 | 1 | IRF8 | 3 |  |  | Crx | 2 | IRF-1 | 2 |
| NKX3-1 | 6 | E4F1 | 1 | GATA2 | 1 | LEF1 | 1 | MAFB | 3 |  |  | MEF-2 | 2 | LBP-1 | 2 |
| STAT1 | 6 | FOXF2 | 1 | LEF1 | 1 | MAFB | 1 | NURR1 | 3 |  |  | PMX2B | 2 | LEF1 | 2 |
| LBP-1 | 5 | Freac-2 | 1 | MEF-2 | 1 | PPARalpha:RXRalpha | 1 | TEF-1 | 3 |  |  | Prop-1 | 2 | NFAT2 | 2 |
| Arnt | 4 | MAX | 1 | MyoD | 1 | PUR1 | 1 | BRCA1 | 2 |  |  | Shox2 | 2 | Nkx3-2 | 2 |
| ATF | 4 | N-Myc | 1 | NRSE | 1 | SOX10 | 1 | Neuro D | 2 |  |  | USF2 | 2 | TEF-1 | 2 |
| IPF1 | 4 | NF-muE1 | 1 | PUR1 | 1 | SPIB | 1 | RFX1 | 2 |  |  | aMEF-2 | 1 | YY1 | 2 |
| MAFB | 4 | NUR77 | 1 | REST | 1 | STAT5A | 1 | USF2 | 2 |  |  | E2A | 1 | CREB | 1 |
| YY1 | 4 | NURR1 | 1 | TBP | 1 | TEAD1 | 1 | AP-3 | 1 |  |  | IRF-1 | 1 | IPF1 | 1 |
| BRCA1 | 3 | Pax-2 | 1 | TEF-1 | 1 | TR4 | 1 | CREB | 1 |  |  | LEF1 | 1 | NURR1 | 1 |
| CLOCK:BMAL | 3 | PITX2 | 1 | YY1 | 1 | YY1 | 1 | CTCF | 1 |  |  | Myc | 1 | PU.1 | 1 |
| FXR | 3 | PPARgamma:RXRalpha | 1 |  |  | ZNF354C | 1 | deltaEF1 | 1 |  |  | Nkx2-5 | 1 | SPIB | 1 |
| Myc | 3 | SPI1 | 1 |  |  |  |  | E4BP4 | 1 |  |  | Otx2 | 1 |  |  |
| AP-2rep | 2 | SRY | 1 |  |  |  |  | En-1 | 1 |  |  | PITX2 | 1 |  |  |
| c-Myc:Max | 2 | Staf | 1 |  |  |  |  | FXR | 1 |  |  | STAT3 | 1 |  |  |
| Crx | 2 | TBP | 1 |  |  |  |  | HSF2 | 1 |  |  | ZNF354C | 1 |  |  |
|  |  | TCF-4 | 1 |  |  |  |  |  |  |  |  |  |  |  |  |

| MSX1 | | | | | | | | | MSX2 | | | | | | | NES | | | | NR4A2 | | | | | | | | | | | | | | | |
| --- | --- | --- | --- | --- | --- | --- | --- | --- | --- | --- | --- | --- | --- | --- | --- | --- | --- | --- | --- | --- | --- | --- | --- | --- | --- | --- | --- | --- | --- | --- | --- | --- | --- | --- | --- |
| HNF4 | 21 | | HoxB5 | | 3 | ICSBP | 1 | | MZF1_4 | | 16 | NFAT2 | | | 2 | MZF1_4 | 10 | | CREB | | | 26 | Myc | | | 3 | Brn-4 | | | 1 | PREP1 | | | 1 |  |
| MZF1_4 | 18 | | IPF1 | | 3 | ISGF-3 | 1 | | MAF | | 10 | Nrf-1 | | | 2 | GATA | 7 | | MZF1_1-4 | | | 18 | NF-kappaB | | | 3 | Cart-1 | | | 1 | PXR (PXR:RXR) | | | 1 |  |
| Dlx-1 | 14 | | MAFB | | 3 | Lhx4 | 1 | | Neuro D | | 10 | REL | | | 2 | CTCF | 5 | | HNF4 | | | 13 | NF-Y | | | 3 | CLOCK:BMAL | | | 1 | RBP-Jkappa | | | 1 |  |
| ETS1 | 11 | | NKX3-1 | | 3 | Mox1 | 1 | | ZNF354C | | 10 | YY1 | | | 2 | TEF | 5 | | ATF | | | 11 | Octamer | | | 3 | Dlx7 | | | 1 | REL | | | 1 |  |
| ZNF354C | 10 | | NURR1 | | 3 | NFAT2 | 1 | | CREB | | 9 | ATF3 | | | 1 | Ets | 4 | | Ets | | | 11 | RP58 | | | 3 | En-1 | | | 1 | RXRA::VDR | | | 1 |  |
| NF-kappaB | 9 | | OTX | | 3 | NFKB1 | 1 | | ETS1 | | 9 | Bach1 | | | 1 | Neuro D | 4 | | GATA3 | | | 8 | YY1 | | | 3 | FOXM1 | | | 1 | SOX9 | | | 1 |  |
| LEF1 | 8 | | c-Rel | | 2 | Nkx2-5 | 1 | | TEF-1 | | 7 | BRCA1 | | | 1 | STAT5A | 4 | | IRF-1 | | | 8 | alpha-CP1 | | | 2 | Gbx2 | | | 1 | SRF | | | 1 |  |
| Neuro D | 8 | | HOXD3 | | 2 | Olf-1 | 1 | | IPF1 | | 6 | c-Rel | | | 1 | HNF4 | 3 | | Nkx3-2 | | | 7 | AP-1 | | | 2 | GCNF | | | 1 | TAL1::TCF3 | | | 1 |  |
| GATA-3 | 7 | | MAFA | | 2 | RelB:p52 (NF-kappaB) | 1 | | STAT5A | | 6 | CRX | | | 1 | En-1 | 2 | | ZNF354C | | | 7 | CHX10 | | | 2 | HNF3A | | | 1 | Tax/CREB | | | 1 |  |
| IRF | 7 | | MECP2 | | 2 | SOX2 | 1 | | GATA | | 5 | DBP | | | 1 | GR | 2 | | LEF1 | | | 6 | CTF1 | | | 2 | HOXC12 | | | 1 | TBX | | | 1 |  |
| STAT1 | 7 | | NF-Y | | 2 | SOX4 | 1 | | IRF-1 | | 5 | Dlx-1 | | | 1 | LBP-1 | 2 | | TBP | | | 6 | FXR | | | 2 | LBP-1 | | | 1 | TCF-4 | | | 1 |  |
| Crx | 6 | | Pitx1 | | 2 | SPI1 | 1 | | Nkx2-5 | | 5 | Elk-1 | | | 1 | MAFB | 2 | | GABP | | | 5 | HNF1 | | | 2 | MEF-2 | | | 1 | TEF-1 | | | 1 |  |
| TEF-1 | 6 | | SRF | | 2 |  |  | | Evi-1 | | 4 | FRA1 | | | 1 | NURR1 | 2 | | HMG IY | | | 5 | lhx6.1 | | | 2 | MIF-1 | | | 1 | VDR:RXR | | | 1 |  |
| GABP-alpha | 5 | | TBP | | 2 |  |  | | LEF1 | | 4 | HNF1 | | | 1 | SPI1 | 2 | | IPF1 | | | 5 | Meis1 | | | 2 | MRG2 | | | 1 | ZBRK1 | | | 1 |  |
| HOXA1 | 5 | | YY1 | | 2 |  |  | | GABP-alpha | | 3 | Lhx5 | | | 1 | BRCA1 | 1 | | OCT01 | | | 5 | N-Myc | | | 2 | MTF1 | | | 1 |  | | |  |  |
| LBP-1 | 5 | | ATF | | 1 |  |  | | NF-Y | | 3 | Mox1 | | | 1 | CRX | 1 | | STAT1 | | | 5 | NF-1 | | | 2 | NFAT2 | | | 1 |  | | |  |  |
| AP-2rep | 4 | | BEN | | 1 |  |  | | SPI-B | | 3 | NF-E2 | | | 1 | deltaEF1 | 1 | | TEF-1 | | | 5 | NKX3-1 | | | 2 | Nkx2-5 | | | 1 |  | | |  |  |
| BRCA1 | 4 | | c-Ets-1 | | 1 |  |  | | AP-1 | | 2 | NFE2L2 | | | 1 | HEN1 | 1 | | c-Myc:Max | | | 4 | PPARG | | | 2 | NURR1 | | | 1 |  | | |  |  |
| CHX10 | 4 | | EBF | | 1 |  |  | | ATF | | 2 | NURR1 | | | 1 | LEF1 | 1 | | CRX | | | 4 | PU.1 | | | 2 | OTX | | | 1 |  | | |  |  |
| REL | 4 | | ER | | 1 |  |  | | FXR | | 2 | RelB:p52 (NF-kappaB) | | | 1 | MAFA | 1 | | Neuro D | | | 4 | SPI1 | | | 2 | P50:RELA-P65 | | | 1 |  | | |  |  |
| SPIB | 4 | | FLI1 | | 1 |  |  | | HMG IY | | 2 | SZF1-1 | | | 1 | Pax-6 | 1 | | SPIB | | | 4 | TLX1::NFIC | | | 2 | Pax-4 | | | 1 |  | | |  |  |
| CREB | 3 | | FOXF2 | | 1 |  |  | | HOXA1 | | 2 | Tax/CREB | | | 1 | PUR1 | 1 | | USF1 | | | 4 | AREB6 | | | 1 | Pit-1 | | | 1 |  | | |  |  |
| DBP | 3 | | Freac-2 | | 1 |  |  | | LBP-1 | | 2 | TBX5 | | | 1 | SOX10 | 1 | | DBP | | | 3 | Bach2 | | | 1 | PKNOX2 | | | 1 |  | | |  |  |
| FXR | 3 | | GR | | 1 |  |  | | LRF | | 2 | TCF-4 | | | 1 | SPIB | 1 | | HOXA7 | | | 3 | BEN | | | 1 | POU2F3 | | | 1 |  | | |  |  |
| HMG IY | 3 | | HOXC-8 | | 1 |  |  | | NF-kappaB | | 2 | Vax-2 | | | 1 | TBP | 1 | | MAFB | | | 3 | BRCA1 | | | 1 | PPARgamma:RXRalpha | | | 1 |  | | |  |  |
|  |  | |  | |  |  |  | |  | |  |  | | |  | TEAD1 | 1 | |  | | |  |  | | |  |  | | |  |  | | |  |  |
|  |  | |  | |  |  |  | |  | |  |  | | |  | YY1 | 1 | |  | | |  |  | | |  |  | | |  |  | | |  |  |
| NEUROD1 | | | | | | | | | | | | | | | | | | | | | OTX2 | | | | | | | | | | | |  |  |  |
| HNF4 | | 18 | | PMX2A | | | | 3 | | Nkx2-5 | | | 2 | HLF | | | | 1 | | | MZF1_1-4 | | | 12 | Octamer | | | 2 | pax6 | | | 1 |  |  |  |
| MZF1_1-4 | | 18 | | PU.1 | | | | 3 | | NRSE | | | 2 | HNF1 | | | | 1 | | | Neuro D | | | 8 | Pax-4 | | | 2 | Pbx1 | | | 1 |  |  |  |
| LEF1 | | 15 | | SRF | | | | 3 | | NRSF | | | 2 | HOXA3 | | | | 1 | | | ZNF354C | | | 7 | PMX2A | | | 2 | POU2F3 | | | 1 |  |  |  |
| USF | | 10 | | TEF-1 | | | | 3 | | RBP-Jkappa | | | 2 | HOXC5 | | | | 1 | | | LEF1 | | | 6 | SRF | | | 2 | PU.1 | | | 1 |  |  |  |
| CRX | | 9 | | AP-1 | | | | 2 | | REL | | | 2 | Lhx4 | | | | 1 | | | LBP-1 | | | 4 | STAT6 | | | 2 | SOX10 | | | 1 |  |  |  |
| c-Myc | | 7 | | Arnt | | | | 2 | | RelB:p52 (NF-kappaB) | | | 2 | Lmx-1 | | | | 1 | | | alpha-CP1 | | | 3 | TEF-1 | | | 2 | SOX4 | | | 1 |  |  |  |
| Ets | | 7 | | Bach1 | | | | 2 | | REST | | | 2 | Msx-1 | | | | 1 | | | HNF4 | | | 3 | USF2 | | | 2 | SPI1 | | | 1 |  |  |  |
| SPI-B | | 6 | | Barx-2 | | | | 2 | | SOX9 | | | 2 | MTF-1 | | | | 1 | | | IRF8 | | | 3 | YY1 | | | 2 | STAT1 | | | 1 |  |  |  |
| AhR | | 5 | | BRCA1 | | | | 2 | | SREBP-1 | | | 2 | MyoD | | | | 1 | | | lhx6.1 | | | 3 | CHX10 | | | 1 | STAT5A | | | 1 |  |  |  |
| MAFA | | 5 | | c-Rel | | | | 2 | | Vax-2 | | | 2 | Nrf-1 | | | | 1 | | | NF-Y | | | 3 | CREB | | | 1 | TRF1 | | | 1 |  |  |  |
| NKX3-1 | | 5 | | CLOCK:BMAL | | | | 2 | | YY1 | | | 2 | PARP | | | | 1 | | | Nkx3-2 | | | 3 | Dlx-1 | | | 1 | Vax-2 | | | 1 |  |  |  |
| STAT3 | | 5 | | CREB | | | | 2 | | Alx-4 | | | 1 | pax6 | | | | 1 | | | Oct01 | | | 3 | EMX2 | | | 1 |  | | |  |  |  |  |
| ZNF354C | | 5 | | CTCF | | | | 2 | | AP-2rep | | | 1 | PMX2B | | | | 1 | | | SPIB | | | 3 | En-1 | | | 1 |  | | |  |  |  |  |
| DBP | | 4 | | E2A | | | | 2 | | Barhl-1 | | | 1 | PNR | | | | 1 | | | ALX-3 | | | 2 | En-2 | | | 1 |  | | |  |  |  |  |
| En-1 | | 4 | | E47 | | | | 2 | | CART1 | | | 1 | PPAR | | | | 1 | | | AML1 | | | 2 | FXR/RXR-alpha | | | 1 |  | | |  |  |  |  |
| FXR | | 4 | | ER | | | | 2 | | CHX10 | | | 1 | RELA | | | | 1 | | | BRCA1 | | | 2 | Gbx2 | | | 1 |  | | |  |  |  |  |
| Max | | 4 | | Esx1 | | | | 2 | | Dlx7 | | | 1 | RXRA::VDR | | | | 1 | | | Esx1 | | | 2 | Helios A | | | 1 |  | | |  |  |  |  |
| NFAT2 | | 4 | | Gfi-1 | | | | 2 | | Ebox | | | 1 | SOX4 | | | | 1 | | | Ets | | | 2 | HOXA3 | | | 1 |  | | |  |  |  |  |
| GABP-alpha | | 3 | | IPF1 | | | | 2 | | Elf-1 | | | 1 | SRY | | | | 1 | | | Evi-1 | | | 2 | IRF-1 | | | 1 |  | | |  |  |  |  |
| GATA3 | | 3 | | IRF-1 | | | | 2 | | EMX2 | | | 1 | TAL1 | | | | 1 | | | Evx-1 | | | 2 | LUN-1 | | | 1 |  | | |  |  |  |  |
| HMG IY | | 3 | | IRF8 | | | | 2 | | Evx-1 | | | 1 | TBX5 | | | | 1 | | | GATA-1 | | | 2 | MAFB | | | 1 |  | | |  |  |  |  |
| HOXA7 | | 3 | | LBP-1 | | | | 2 | | FRA1 | | | 1 | TCF-4 | | | | 1 | | | HB9 | | | 2 | Msx-1 | | | 1 |  | | |  |  |  |  |
| lhx6.1 | | 3 | | N-Myc | | | | 2 | | HB9 | | | 1 | Whn | | | | 1 | | | HOXA7 | | | 2 | Myc | | | 1 |  | | |  |  |  |  |
| Myc | | 3 | | Neuro D | | | | 2 | | hbp1 | | | 1 |  | | | |  | | | IPF1 | | | 2 | NF-muE1 | | | 1 |  | | |  |  |  |  |
| NURR1 | | 3 | | NF-kappaB | | | | 2 | | HEB | | | 1 |  | | | |  | | | LH-2 | | | 2 | NURR1 | | | 1 |  | | |  |  |  |  |

| PAX6 | | | | PITX3 | | | | S100B | | |  | SOX6 | | | | | |
| --- | --- | --- | --- | --- | --- | --- | --- | --- | --- | --- | --- | --- | --- | --- | --- | --- | --- |
| MZF1_1-4 | 9 | FOXC1 | 1 | Neuro D | 14 | CRX | 1 | MZF1_1-4 | 8 | LBP-1 | 1 | IPF1 | 7 | Lhx3 | 2 | HOXC4 | 1 |
| CREB | 8 | Freac-2 | 1 | HNF4 | 7 | DBP | 1 | HNF4 | 6 | NERF1a | 1 | GATA | 5 | Lhx4 | 2 | HOXC5 | 1 |
| ATF | 4 | GABP-alpha | 1 | USF | 6 | ETS2 | 1 | LEF1 | 6 | NF-E2 | 1 | IRF-1 | 5 | Lhx5 | 2 | LH-2 | 1 |
| GLI1 | 4 | GATA | 1 | LBP-1 | 5 | INSM1 | 1 | Neuro D | 6 | NF-Y | 1 | OCT01 | 5 | PMX2A | 2 | Lmx-1 | 1 |
| c-Myc:Max | 3 | GR | 1 | MZF1_1-4 | 5 | IPF1 | 1 | Ets | 5 | NURR1 | 1 | CRX | 4 | PMX2B | 2 | LUN-1 | 1 |
| Crx | 3 | IRF-1 | 1 | c-Myc:Max | 3 | IRF-1 | 1 | TEF-1 | 5 | OCT01 | 1 | lhx6.1 | 4 | POU6F1 | 2 | MAFB | 1 |
| STAT1 | 3 | LBP-1 | 1 | GATA2 | 3 | ISGF-3 | 1 | MAFA | 4 | SPI1 | 1 | RFX1 | 4 | Shox2 | 2 | MEF-2 | 1 |
| AR | 2 | MAFA | 1 | MIZF | 3 | LEF1 | 1 | Nkx3-2 | 4 | TBP | 1 | Brn-4 | 3 | SOX9 | 2 | OCT-x | 1 |
| ATF2:c-Jun | 2 | mTERF | 1 | NF-Y | 3 | LUN-1 | 1 | IPF1 | 3 | Tel-2 | 1 | CART1 | 3 | Barhl-1 | 1 | OTX2 | 1 |
| BRCA1 | 2 | MTF1 | 1 | NURR1 | 3 | MAFA | 1 | MAFB | 3 |  |  | CHX10 | 3 | Brachyury | 1 | Pax-4 | 1 |
| LEF1 | 2 | N-Myc | 1 | SPIB | 3 | MAX | 1 | AP-2rep | 2 |  |  | Evi-1 | 3 | CDP | 1 | POU2F3 | 1 |
| MAFB | 2 | NF-Y | 1 | BEN | 2 | MYB | 1 | OTX | 2 |  |  | Gbx2 | 3 | DMRT2 | 1 | rax | 1 |
| Myc | 2 | Nkx2-5 | 1 | CLOCK:BMAL | 2 | NF-muE1 | 1 | SOX10 | 2 |  |  | HNF4 | 3 | DMRT3 | 1 | RORalpha1 | 1 |
| Neuro D | 2 | OCT-x | 1 | Myc | 2 | Nkx3-2 | 1 | STAT5A | 2 |  |  | MZF1_1-4 | 3 | EMX2 | 1 | RORBETA | 1 |
| Nkx3-2 | 2 | Octamer | 1 | MZF1_1-4 | 2 | PU.1 | 1 | STAT6 | 2 |  |  | Octamer | 3 | En-1 | 1 | SOX10 | 1 |
| Oct01 | 2 | Pax-5 | 1 | N-Myc | 2 | PXR (PXR:RXR) | 1 | YY1 | 2 |  |  | Pit-1 | 3 | En-2 | 1 | SPI1 | 1 |
| USF | 2 | PITX3 | 1 | SMAD | 2 | TEF-1 | 1 | ZNF354C | 2 |  |  | ALX-3 | 2 | GCNF | 1 | TBP | 1 |
| USF2 | 2 | POU2F3 | 1 | STAT5A | 2 |  |  | alpha-CP1 | 1 |  |  | Alx-4 | 2 | Gfi1b | 1 | TBX18 | 1 |
| AP-2rep | 1 | PXR (PXR:RXR) | 1 | YY1 | 2 |  |  | AP-1 | 1 |  |  | Barhl2 | 2 | GZF1 | 1 | TEF-1 | 1 |
| AR | 1 | REST | 1 | ZNF354C | 2 |  |  | BRCA1 | 1 |  |  | Dlx-1 | 2 | HMG IY | 1 | Vax-2 | 1 |
| ATF3 | 1 | SOX10 | 1 | AML1 | 1 |  |  | c-Ets-1 | 1 |  |  | Esx1 | 2 | HMX1 | 1 |  |  |
| CLOCK:BMAL | 1 | SPIB | 1 | AP-2rep | 1 |  |  | CREB | 1 |  |  | HB9 | 2 | HNF-1beta | 1 |  |  |
| Dlx-2 | 1 | SRF | 1 | AP-4 | 1 |  |  | CRX | 1 |  |  | HOXC10 | 2 | HOXA1 | 1 |  |  |
| Ebox | 1 | TEF-1 | 1 | BRCA1 | 1 |  |  | FXR | 1 |  |  | HOXC11 | 2 | HOXA3 | 1 |  |  |
| ETS1 | 1 | ZNF354C | 1 | CREB | 1 |  |  | IRF8 | 1 |  |  | K-2b | 2 | HOXA4 | 1 |  |  |

| TH | | | | NR0B1 | | |  | NR0B2 | | | | WNT1 | | | |
| --- | --- | --- | --- | --- | --- | --- | --- | --- | --- | --- | --- | --- | --- | --- | --- |
| MZF1_1-4 | 22 | GATA2 | 1 | IPF1 | 9 | Nrf-1 | 2 | HNF4 | 9 | HOXA1 | 1 | CREB | 9 | ELK4 | 1 |
| HNF4 | 18 | IRF-1 | 1 | LEF1 | 8 | NURR1 | 2 | USF | 8 | HoxA2 | 1 | ETS1 | 9 | En-1 | 1 |
| Neuro D | 8 | LBP-1 | 1 | Neuro D | 6 | AhR, Arnt, HIF-1 | 1 | MZF1 | 6 | HOXA5 | 1 | HNF4 | 9 | HEN1 | 1 |
| ZNF354C | 8 | Msx-2 | 1 | NKX3-1 | 6 | Bach1 | 1 | Myc | 4 | HOXA7 | 1 | STAT1 | 6 | HMG IY | 1 |
| ATF | 7 | NFAT2 | 1 | STAT3 | 6 | c-Myc:Max | 1 | Neuro D | 4 | HoxB5 | 1 | GABP-alpha | 5 | IRF-1 | 1 |
| MAFB | 7 | NURR1 | 1 | MAFA | 5 | E47 | 1 | ZNF354C | 3 | HOXC9 | 1 | MZF1_1-4 | 5 | MyoD | 1 |
| PUR1 | 7 | SOX10 | 1 | TEF-1 | 5 | ERR2 (ESRRB) | 1 | AP-1 | 2 | HOXD3 | 1 | MAFB | 4 | MZF1 | 1 |
| CREB | 6 |  |  | AP-1 | 4 | FRA1 | 1 | c-Myc:Max | 2 | ipf1 | 1 | NF-kappaB | 4 | NERF1a | 1 |
| YY1 | 6 |  |  | ETS1 | 4 | GATA | 1 | CLOCK:BMAL | 2 | KAISO | 1 | ATF | 3 | NGFI-C | 1 |
| STAT3 | 5 |  |  | HMG IY | 4 | GR | 1 | LEF1 | 2 | LBP-1 | 1 | CTCF | 3 | Nkx3-2 | 1 |
| LEF1 | 3 |  |  | IRF-1 | 4 | HEB | 1 | MAFA | 2 | MAFB | 1 | GLI1 | 3 | NURR1 | 1 |
| TEF-1 | 3 |  |  | SF1 | 4 | HIF1 | 1 | N-Myc | 2 | MAX | 1 | LEF1 | 3 | RBP-Jkappa | 1 |
| USF2 | 3 |  |  | SPIB | 4 | LBP-1 | 1 | AP-2rep | 1 | Mox1 | 1 | ZNF354C | 3 | RELA | 1 |
| AP-2rep | 2 |  |  | DBP | 3 | MECP2 | 1 | Bach2 | 1 | MTF1 | 1 | AML1 | 2 | RORBETA | 1 |
| CRX | 2 |  |  | IRF8 | 3 | MEIS1 | 1 | E12 | 1 | Nkx2-5 | 1 | BRCA1 | 2 | RXR::RAR_DR5 | 1 |
| E2A | 2 |  |  | Nkx2-5 | 3 | Meis2 | 1 | E2A | 1 | Nkx3-2 | 1 | DBP | 2 | SPIB | 1 |
| ETS2 | 2 |  |  | TCF-4 | 3 | MyoD | 1 | En-1 | 1 | NURR1 | 1 | GATA2 | 2 | SREBP1 | 1 |
| IPF1 | 2 |  |  | YY1 | 3 | NF-E2 | 1 | ESR1 | 1 | RORA_1 | 1 | INSM1 | 2 | SZF1-1 | 1 |
| MAFA | 2 |  |  | AhR | 2 | PU.1 | 1 | ETS2 | 1 | RORalpha1 | 1 | IRF8 | 2 | Tax/CREB | 1 |
| Nkx3-2 | 2 |  |  | Bach2 | 2 | TBP | 1 | FRA1 | 1 | SMAD | 1 | Neuro D | 2 | TEF-1 | 1 |
| BEN | 1 |  |  | CRX | 2 | TFE | 1 | FXR inverted repeat 1 | 1 | SPIB | 1 | AML | 1 | TRF1 | 1 |
| BRCA1 | 1 |  |  | EBF | 2 | ZNF354C | 1 | FXR/RXR-alpha | 1 | TAL1 | 1 | c-Ets-1(p54) | 1 | YY1 | 1 |
| CHX10 | 1 |  |  | GABP-alpha | 2 |  |  | GATA2 | 1 | TEF-1 | 1 | c-Myc:Max | 1 | ZBRK1 | 1 |
| DBP | 1 |  |  | MZF1_1-4 | 2 |  |  | hbp1 | 1 | YY1 | 1 | E2F1 | 1 |  |  |
| FXR | 1 |  |  | NFAT2 | 2 |  |  | HEB | 1 |  |  | Egr-3 | 1 |  |  |

| WNT5A | | | | | | | | RSPO2 | | | |
| --- | --- | --- | --- | --- | --- | --- | --- | --- | --- | --- | --- |
| MZF1_1-4 | 23 | AP-2rep | 2 | Egr-3 | 1 | PMX2A | 1 | MZF1_1-4 | 9 | ETS2 | 1 |
| HNF4 | 14 | Cdx-2 | 2 | EMX2 | 1 | rax | 1 | GATA | 7 | FXR | 1 |
| ZNF354C | 11 | CHX10 | 2 | En-2 | 1 | RelB:p52 (NF-kappaB) | 1 | LBP-1 | 6 | FXR/RXR-alpha | 1 |
| LEF1 | 10 | CRX | 2 | Evx-1 | 1 | RXR::RAR_DR5 | 1 | LEF1 | 6 | GR | 1 |
| STAT1 | 9 | CTF1 | 2 | FOXD1 | 1 | TAL1 | 1 | Neuro D | 6 | HMG IY | 1 |
| Neuro D | 7 | DBP | 2 | FRA1 | 1 | TCF-4 | 1 | USF | 6 | HSF | 1 |
| IPF1 | 6 | dlx5 | 2 | GABP-alpha | 1 | ZID | 1 | IRF-1 | 4 | IPF1 | 1 |
| Nkx3-2 | 6 | Gbx2 | 2 | GCM | 1 |  |  | TEF | 4 | LH-2 | 1 |
| TEF-1 | 6 | HB9 | 2 | HIC1 | 1 |  |  | ZNF354C | 4 | MAFA | 1 |
| GATA | 5 | HOXA1 | 2 | HMG IY | 1 |  |  | Myc | 3 | MAFB | 1 |
| YY1 | 5 | HOXB9 | 2 | HoxA2 | 1 |  |  | SPI1 | 3 | N-Myc | 1 |
| En-1 | 4 | NFAT2 | 2 | HOXA3 | 1 |  |  | Evi-1 | 2 | NF-kappaB (p65) | 1 |
| IRF-1 | 4 | Nrf-1 | 2 | hoxa9 | 1 |  |  | MTF1 | 2 | NKX3-1 | 1 |
| MAFB | 4 | TLX1::NFIC | 2 | HoxB5 | 1 |  |  | Nkx2-5 | 2 | NURR1 | 1 |
| SPIB | 4 | Vax-2 | 2 | HOXC9 | 1 |  |  | Nkx3-2 | 2 | OTX2 | 1 |
| SRF | 4 | Alx-4 | 1 | HSF2 | 1 |  |  | Pax-2 | 2 | PU.1 | 1 |
| BRCA1 | 3 | AREB6 | 1 | ISL1 | 1 |  |  | TCF-4 | 2 | REL | 1 |
| ETS1 | 3 | Cdx-1 | 1 | lhx6.1 | 1 |  |  | ALX-3 | 1 | RORA_1 | 1 |
| ETS2 | 3 | CLOCK:BMAL | 1 | LXR | 1 |  |  | AP-2rep | 1 | SF1 | 1 |
| GABP-alpha | 3 | CREB | 1 | MTF1 | 1 |  |  | Barhl2 | 1 | SPIB | 1 |
| LBP-1 | 3 | deltaEF1 | 1 | MYC::MAX | 1 |  |  | c-Rel | 1 | SRY | 1 |
| MAFA | 3 | Dlx-1 | 1 | Nkx2-5 | 1 |  |  | CLOCK:BMAL | 1 | STAT1:STAT1 | 1 |
| NF-1 | 3 | dlx3 | 1 | NKX3-1 | 1 |  |  | CREB | 1 | STAT5A | 1 |
| USF1 | 3 | Dlx7 | 1 | NURR1 | 1 |  |  | CRX | 1 | TBP | 1 |
| AP-1 | 2 | E2F1 | 1 | Pbx-1b | 1 |  |  | DBP | 1 | TEAD1 | 1 |
|  |  |  |  |  |  |  |  |  |  | YY1 | 1 |
